# A Randomized Controlled Study of the Yi Qi Gu Biao Pill in the Treatment of Frequent Exacerbator Phenotype in Chronic Obstructive Pulmonary Disease (Lung and Spleen Qi Deficiency Syndrome)

**DOI:** 10.1155/2017/9130804

**Published:** 2017-12-04

**Authors:** Gao Zhen, Jing Jing, Xu Dan, Li Zheng, Li Fengsen, Sun Qi

**Affiliations:** National Clinical Research Base of Traditional Chinese Medicine, Traditional Chinese Medicine Hospital Affiliated to Xinjiang Medical University, Urumqi 830000, China

## Abstract

**Objective:**

To evaluate the efficacy and safety of Yi Qi Gu Biao (YQGB) pill in treating frequent exacerbator phenotype in chronic obstructive pulmonary disease (lung and spleen qi deficiency syndrome) (FEPCOPD).

**Methods:**

This prospective, randomized, double-blind, controlled study assessed 112 cases (64 included) of FEPCOPD treated at the outpatient department in our hospital in January–August 2016. The patients were randomly divided into YQGB and placebo (Pb) and treated for three months. Lung function, CAT, mMRC, and TCM symptom scores (TCMs) were observed.

**Results:**

Compared with Pb, YQGB showed decreased wheezing symptom scores (WSs) and TCMs at one month and decreased CAT and TCMs at three months. From one to three months, CAT, cough, sputum, WSs, and TCMs in YQGB were lower than pretreatment values. But in Pb, CAT was lower than pretreatment values after one month; CAT, sputum, and TCMs were lower than pretreatment values after two months; CAT, cough, sputum, WSs, and TCMs were lower than pretreatment values after three months.

**Conclusion:**

Yi Qi Gu Biao pill can improve wheezing, health status, and TCMs in FEPCOPD and also can shorten the durations of cough, sputum, and wheezing. This trial is registered in the Clinical Trials Registry of China: ChiCTR-IOR-15007542 (on 8 December 2015).

## 1. Introduction

The clinical phenotype refers to disease characteristics associated with clinical manifestations such as symptoms, acute exacerbation, treatment response, disease progression, and death that can reflect the differences among COPD patients [1] and has constituted a hot research topic for chronic obstructive pulmonary disease (COPD) in recent years. Phenotypic identification not only helps understand the differences between COPD types, but also more importantly has potential diagnostic and therapeutic significance; in addition, it can better reflect the heterogeneity of COPD, prompting a more comprehensive and in-depth research in COPD. According to the relationship between the clinical course of COPD and prognosis, as well as the different responses of currently available treatment methods, three phenotypes can be distinguished, including frequent exacerbator phenotype, COPD-asthma overlap, and emphysema-airway hyperresponsiveness [2]. The frequent exacerbator phenotype is the most frequently encountered in COPD patients; it occurs not only in the severe phase of COPD, but also in 22% of patients with GOLD grade 2 disease [3]. The frequent exacerbator phenotype in COPD refers to patients with more than two yearly disease episodes, with oral administration of glucocorticoids and/or antibiotics needed each time, or those requiring hospitalization [1]. The pathophysiology underlying the frequent exacerbator phenotype is complex, with increased airway and systemic inflammation, dynamic lung hyperinflation, changes in the bacterial colonization lower airways, and a possible increased susceptibility to viral infections. Patients with the frequent exacerbator phenotype are also at increased risk of comorbid extrapulmonary diseases, including cardiovascular disease, gastroesophageal reflux, depression, osteoporosis, and cognitive impairment. Overall, these patients have poorer health status, accelerated loss of forced expiratory volume over 1s (FEV1), worsened quality of life, and increased hospital admissions and mortality, contributing to increased exacerbation susceptibility and perpetuation of the frequent exacerbator phenotype [4]. Therefore, the frequent exacerbator phenotype in COPD is well worthy of special attention from clinicians and scientists.

A previous research demonstrated that [5] Yi Qi Gu Biao pill can prolong the interval of acute exacerbation in COPD patients by 77.68 days, while reducing the number of acute exacerbations by 1.42/6 months. Moreover, its therapeutic effects are consistent with the main treatment purpose for the frequent exacerbator phenotype in COPD, that is, reduction of the number of acute exacerbations. Therefore, this randomized controlled study assessed the efficacy and safety of Yi Qi Gu Biao pill in treating the frequent exacerbator phenotype in COPD (lung and spleen qi deficiency syndrome) [6].

## 2. Materials and Methods

### 2.1. Diagnostic Criteria

The diagnostic criteria of COPD were based on* “the Global Strategy for Diagnosis, Treatment, and Prevention of Chronic Obstructive Pulmonary Diseases (Revised Edition in 2011)”* [7]. The diagnostic criteria for the frequent exacerbator phenotype in COPD were [1, 3] more than two yearly disease onset events, requiring oral administration of glucocorticoids and/or antibiotics and requirement for hospitalization during treatment. Three years were used as a prospective endpoint.

### 2.2. TCM Diagnosis and Criteria of Syndrome Differential Classification

Diagnostic criteria for lung and spleen qi deficiency syndrome were based on the “*diagnostic criteria of TCM syndromes for chronic obstructive pulmonary disease (2011 revision)*” proposed by the Committee of Chinese Medicine Association of Internal Medicine Branch of the Pulmonary Disease [8].

### 2.3. Inclusion Criteria

The following inclusion criteria were adopted: (1) diagnostic criteria for chronic obstructive pulmonary disease at a stable stage; (2) diagnostic criteria of TCM syndrome of qi deficiency of lung and spleen; (3) age between 40 and 75 years; (4) survival period greater than three months; (5) good compliance, agreement to cooperate in this study, and signed informed consent.

### 2.4. Exclusion Criteria

Patients were excluded due to the following: (1) comorbidity with diseases such as bronchial asthma, tuberculosis, bronchiectasis, pulmonary cystic fibrosis, lung cancer, lung abscess, and congestive heart failure; (2) combined primary diseases such as severe cardiovascular and cerebrovascular diseases, liver and kidney ailments, and hematopoietic system diseases; (3) pregnant or lactating women; (4) mental diseases; (5) disability such as blindness, deafness, mental retardation, and physical impairment; (6) allergy or contraindications to the experimental drugs; (7) combined with tumors; (8) congenital or acquired immunodeficiency; and (9) implication in other clinical experiments within the past month.

### 2.5. Withdrawal Criteria

The following were considered: (1) patient change of medical prescription half-way or supplementation of a combination of nonprescribed drugs, especially those that have large impact on the experimental drugs, affecting judgment of effectiveness and safety; (2) development of allergic reactions or serious adverse events; (3) other complications and special physiological changes disqualifying from further testing; and (4) voluntary withdrawal.

### 2.6. Administration Method [5]

The YQGB group was treated with the drug during stable stage as recommended by “*the Global Strategy for the Diagnosis, Treatment, and Prevention of Chronic Obstructive Pulmonary Disease (2011 revision).*” Meanwhile, oral administration of the Yi Qi Gu Biao pill (10 pills/time and 0.19 g/pill) was performed three times every day with a total treatment course of 12 weeks. The Pb group was treated based on the “*the Global Strategy for the Diagnosis, Treatment, and Prevention of Chronic Obstructive Pulmonary Disease (the 2011 revision).*” Meanwhile, oral administration of the placebo (10 pills/time and 0.19 g/pill) was performed three times every day with a total treatment course of 12 weeks.

### 2.7. Observation Outcomes and Methods

#### 2.7.1. Pulmonary Function Test

The pulmonary function of patients was tested on a HI-101 pulmonary function instrument manufactured by CHEST Co., Ltd., Japan. Before the test, the investigators first explained the specific operational procedures and requirements to the subjects to ensure accurate and reliable results. All operations were completed by the same technician, and the following pulmonary function indicators were detected: forced expiratory volume in one second (FEV1) and FEV1 percentage in the predicted value (FEV1% pred), forced vital capacity (FVC) and FVC percentage in the predicted value (FVC% pred), and FEV1/FVC.

#### 2.7.2. Assessment Methods and Standards of COPD Assessment Test (CAT)

The Chinese version of the CAT scoring questionnaire was used [9]. The CAT questionnaire comprises a total of eight questions, including cough, sputum, chest tightness, the feeling of climbing or climbing a staircase, housework, degree of confidence away from home, sleep, and energy. The score for each question from mild to severe disease was 0–5 points; the total score was calculated after scoring each question by the patients, and CAT scores ranged between 0 and 40 points. Upon definite diagnosis, the same researcher explained the questionnaire contents and the scoring method to the patients. The patients filled the questionnaire independently without reminder. Finally, two investigators evaluated the scores.

#### 2.7.3. Classification Criteria of Modified Medical British Research Council (mMRC) [10]

The disease was graded as follows: 0, dyspnea occurring only during exercise; 1, shortness of breath only after fast walking on a flat surface or walking up a small slope; 2, due to shortness of breath, slower walking on a flat surface than the peers or need to stop for rest; 3, stop breathing after walking for about 100 meters on a flat surface or a few minutes; 4, inability to leave home due to severe dyspnea, or dyspnea occurring during dressing and undressing.

#### 2.7.4. TCM Syndrome Score

The TCM syndrome score was based on the quantitative criteria of chronic bronchitis symptom standards in “*Guidelines for Clinical Research of Chinese Drugs,*” including the eight syndromes of cough, sputum, wheezing, weight loss, fatigue, poor appetite, abdominal distension, and loose stool.

### 2.8. Statistical Methods

Group-wise comparison between the YQGB and Pb groups was performed by independent samples* t*-test; comparison before and after the treatment for each group was assessed by paired* t-*test. Chi-square test was used for categorical variables. For the CAT, the minimally important difference is generally accepted as a 2-point change, so chi-square test was used for categorical data ≥2 points and <2 points. A *P* value of <0.05 was deemed statistically significant. All data were analyzed with SPSS version 17.0 (IBM Corp., Armonk, NY, USA).

## 3. Results

### 3.1. General Data

The 112 subjects were follow-up visit patients at the National Clinical Research Base of Traditional Chinese Medicine of the Affiliated TCM Hospital of Xinjiang Medical University, between January 2016 and August 2016. A randomized, double-bind, single-treatment, and parallel controlled experimental design was used. The patients were divided into the YQGB and Pb groups, with each group involving 56 patients. After exclusion, there were 33 patients in the YQGB group and 31 in the Pb group. There were no statistically significant differences in gender, height, weight, age, BMI, pulmonary function, CAT, mMRC, and TCM syndrome score (Table 1) between the two groups of patients.

### 3.2. Group-Wise Comparison

#### 3.2.1. Pulmonary Functions between YQGB and Pb Groups

**Figure 1:**
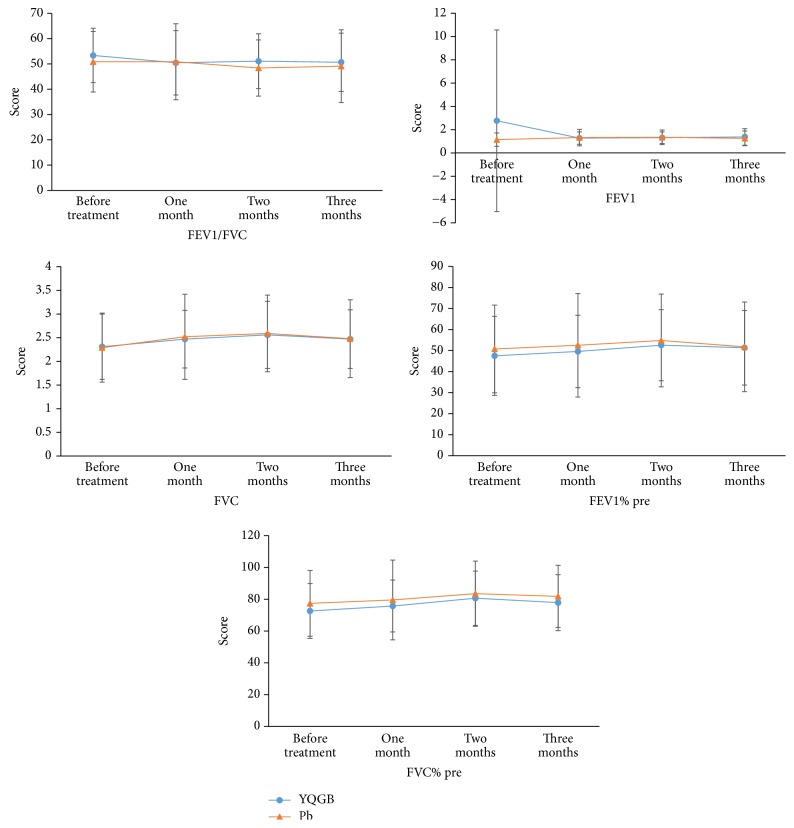
Pulmonary functions in the two groups in three months of treatment.

There were no statistical significant differences in pulmonary functions between the YQGB and Pb groups, within the three months of treatment (Figure 1).

#### 3.2.2. CAT, mMRC, and TCM Syndrome Scores

**Figure 2:**
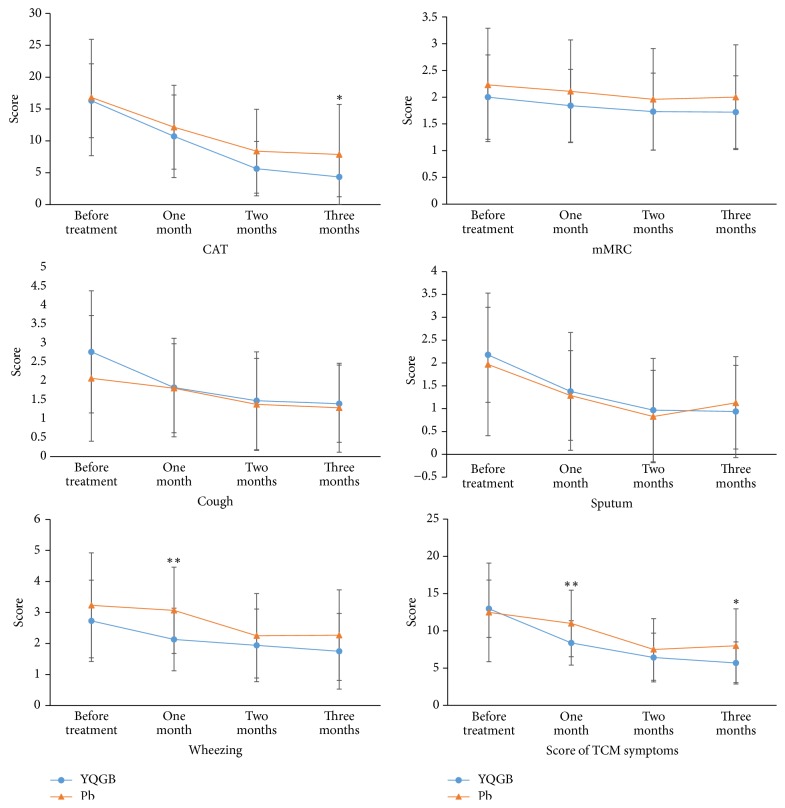
CAT, mMRC, and TCM syndrome scores in the two groups in three months of treatment. ^*∗*^*P* < 0.05, ^*∗∗*^*P* < 0.01.

Between the YQGB and Pb groups, the YQGB group showed a reduced score of wheezing symptom at one month compared with the Pb group (*P* < 0.01); TCM syndrome score was reduced as well (*P* < 0.01). The YQGB group showed decreased CAT (*P* < 0.05) and TCM syndrome (*P* < 0.05) scores at three months compared with the Pb group (Figure 2, Table 2).

#### 3.2.3. Pulmonary Functions before and after the Treatment between the YQGB and Pb Groups

**Figure 3:**
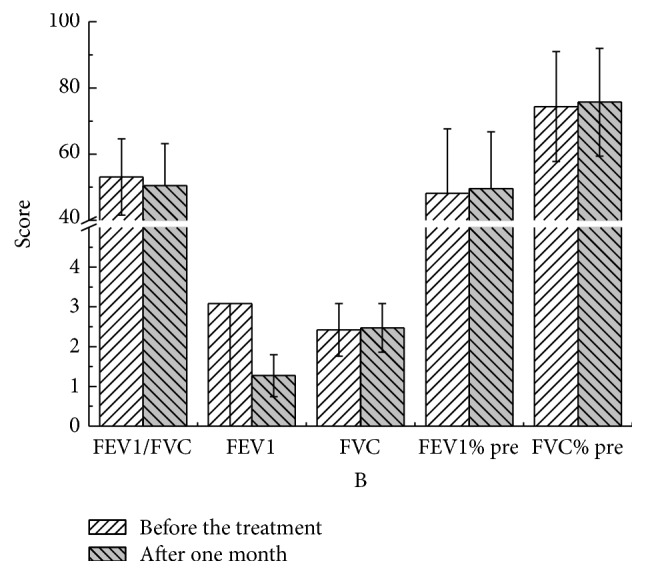
Pulmonary functions in the YQGB group after one month and before treatment.

**Figure 4:**
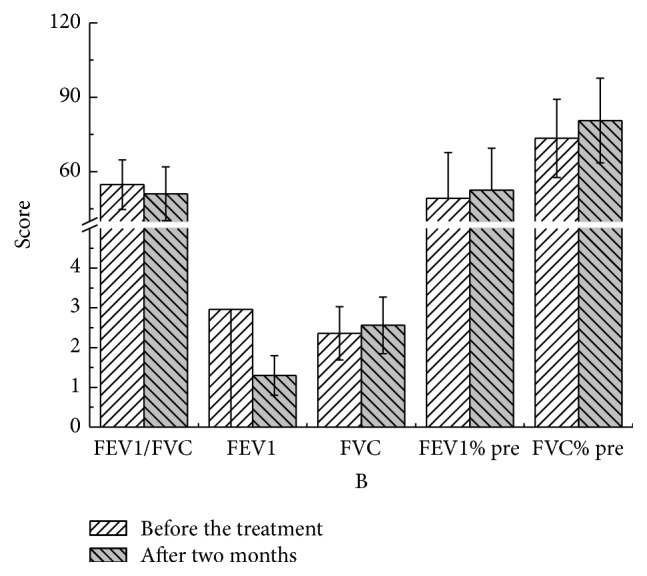
Pulmonary functions in the YQGB group after two months and before the treatment.

**Figure 5:**
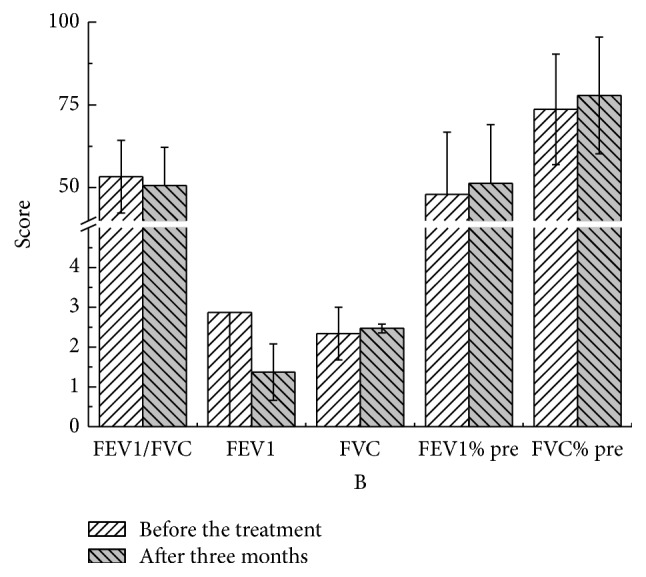
Pulmonary functions in the YQGB group after three months and before the treatment.

**Figure 6:**
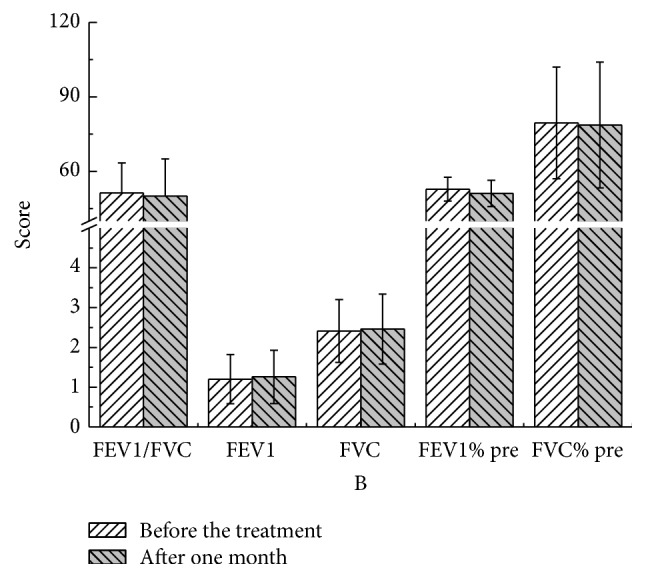
Pulmonary functions in the Pb group after one month and before the treatment.

**Figure 7:**
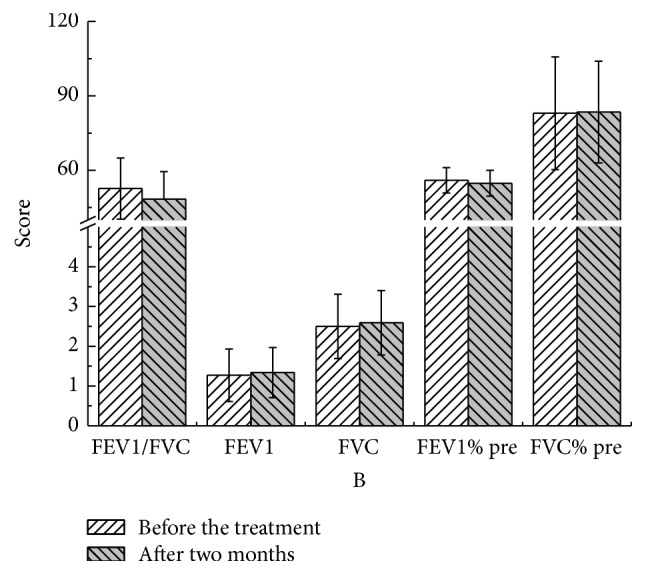
Pulmonary functions in the Pb group after two months and before the treatment.

**Figure 8:**
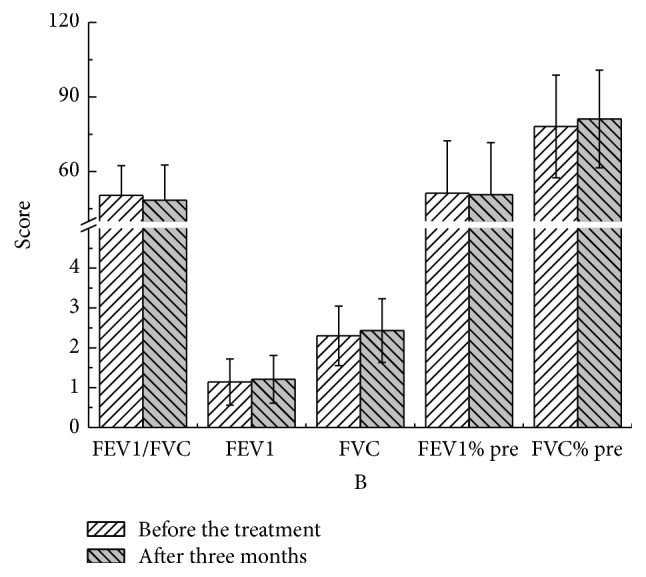
Pulmonary functions in the Pb group after three months and before the treatment.

There were no statistical significant differences in these parameters at different time points compared to pretreatment values in both YQGB group and Pb group (Figures 3–8).

#### 3.2.4. CAT, mMRC, Main Symptom, and TCM Syndrome Scores before and after the Treatment between the YQGB and Pb Groups

**Figure 9:**
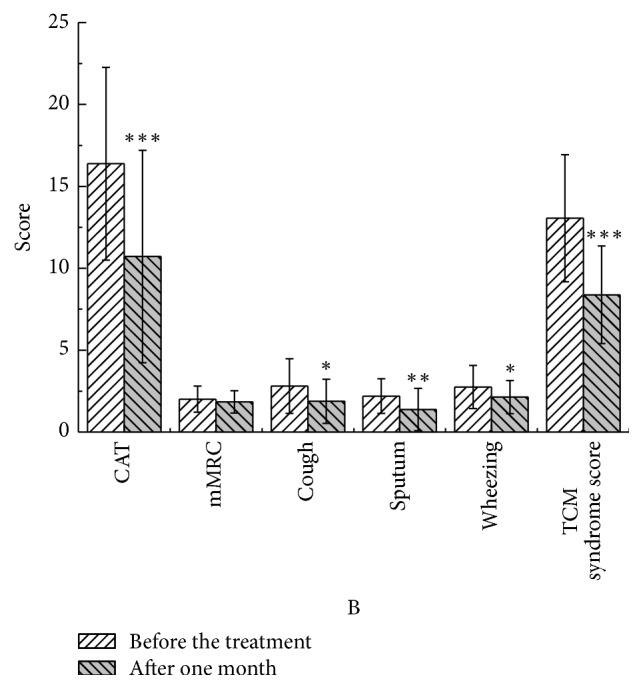
CAT and mMRC scores in the YQGB group after one month and before the treatment. ^*∗*^*P* < 0.05, ^*∗∗*^*P* < 0.01, and ^*∗∗∗*^*P* < 0.001.

**Figure 10:**
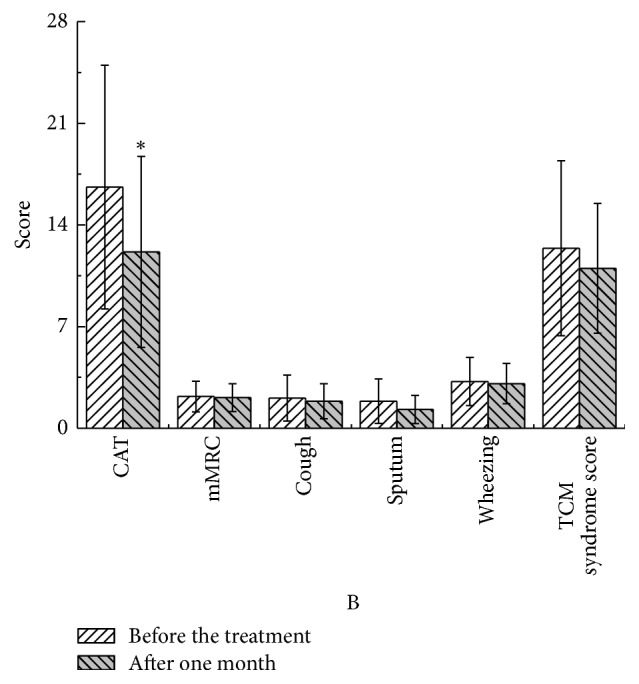
CAT and mMRC in the Pb group after one month and before the treatment. ^*∗*^*P* < 0.05.

After one month, in the YQGB group CAT scores were lower than pretreatment values (*P* < 0.01); cough, sputum, and wheezing symptom scores were also lower (*P* < 0.05), as well as TCM syndrome scores (*P* < 0.01). In the Pb group, CAT was lower than pretreatment values (*P* < 0.05) (Figures 9 and 10).

**Figure 11:**
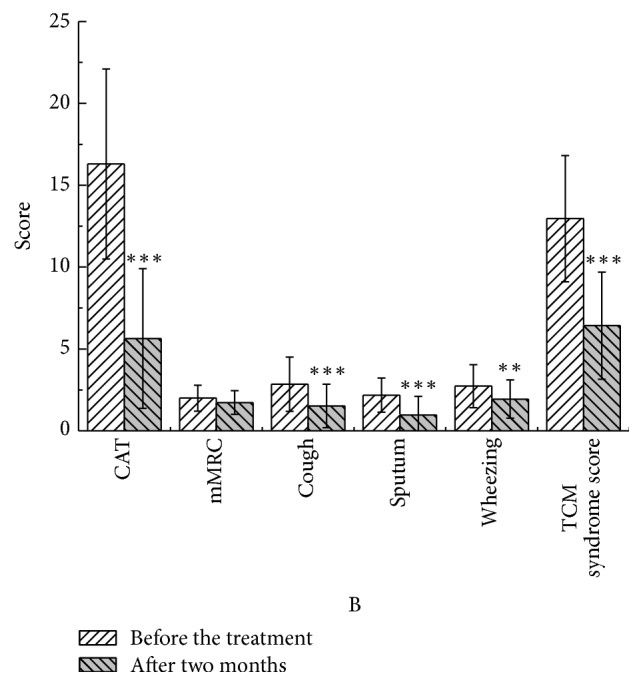
CAT and mMRC in the YQGB group after two months and before the treatment. ^*∗∗*^*P* < 0.01, ^*∗∗∗*^*P* < 0.001.

**Figure 12:**
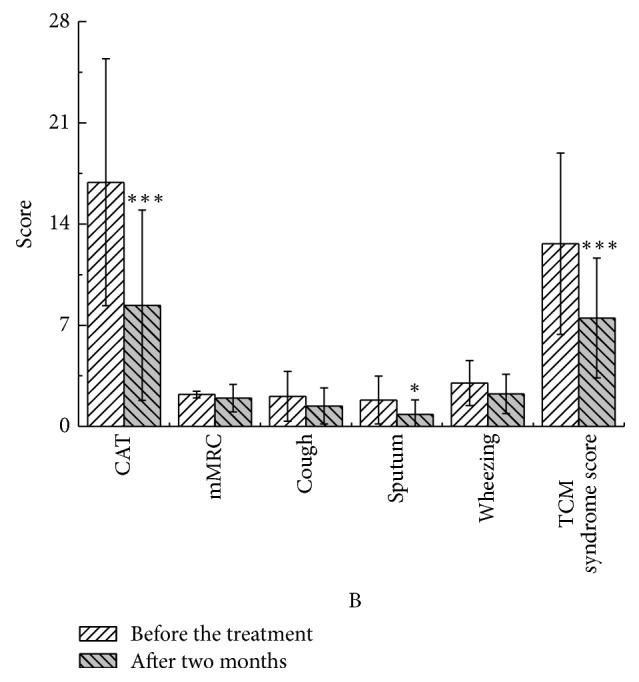
CAT and mMRC in the Pb group after two months and before the treatment. ^*∗*^*P* < 0.05, ^*∗∗∗*^*P* < 0.001.

After two months, in the YQGB group, CAT scores were lower than pretreatment values (*P* < 0.01), as well as cough, sputum, and wheezing symptom scores (*P* < 0.01, *P* < 0.01, and *P* < 0.05), and TCM syndrome scores (*P* < 0.01). In the Pb group, only CAT and sputum symptom scores were lower than pretreatment values (*P* < 0.01, *P* < 0.05) (Figures 11 and 12).

**Figure 13:**
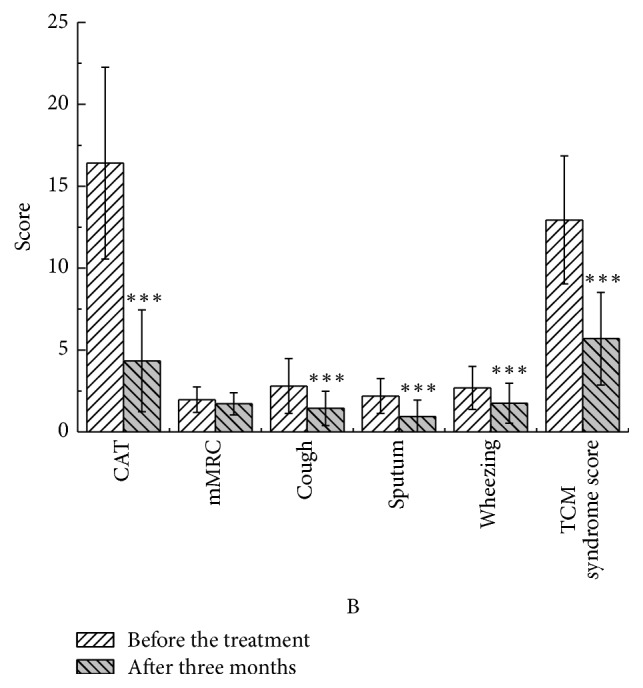
CAT and mMRC in the YQGB group after three months and before the treatment. ^*∗∗∗*^*P* < 0.001.

**Figure 14:**
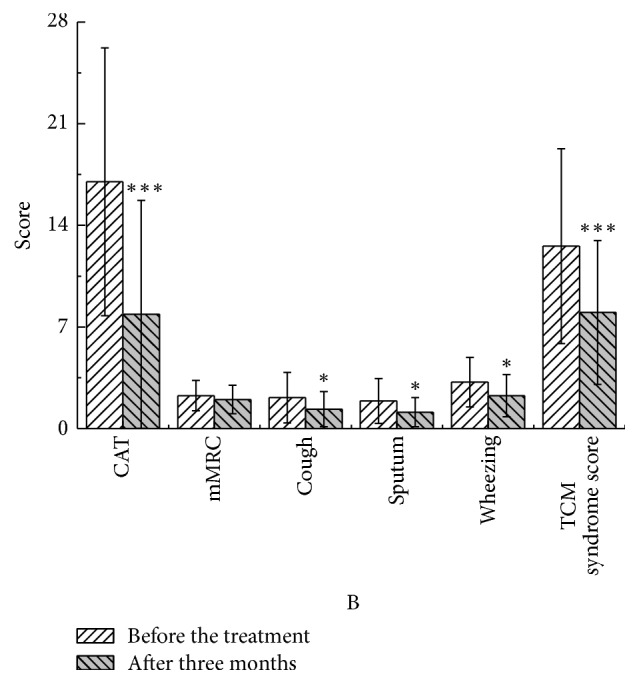
CAT and mMRC in the Pb group after three months and before the treatment. ^*∗*^*P* < 0.05, ^*∗∗∗*^*P* < 0.001.

After three months, in the YQGB group, CAT scores were lower than pretreatment values (*P* < 0.01), as well as cough, sputum, and wheezing symptom scores (*P* < 0.01) and TCM syndrome scores (*P* < 0.01). In the Pb group, CAT scores were lower than pretreatment values (*P* < 0.01), as well as cough, sputum, and wheezing symptom scores (*P* < 0.05) and TCM syndrome scores (*P* < 0.01) (Figures 13 and 14).

### 3.3. Adverse Reactions

There were no abnormal changes of clinical significance in laboratory indicators such as blood and urine routine tests, renal function, and ECG. Moreover, there were no adverse reactions such as nausea, abdominal pain, and diarrhea.

## 4. Discussion

COPD prevalence is 8.2% in the subpopulation above 40 years old in the seven provinces in China [11] and 13.4% in Korea [12]. COPD is an important cause of disability and death worldwide, imposing huge and growing economic and social burden to mankind [13]. Therefore, how to effectively control COPD has become a major public health problem that needs urgent solution. COPD is a disorder of progressive airflow limitation caused by chronic inflammation of the airways and lung parenchyma; it is associated with symptoms such as cough, sputum production, and dyspnea [14]. However, the risk of acute exacerbation in COPD patients with symptoms of chronic cough and sputum is increased by 4.15 times, with the risk of hospitalization due to acute exacerbation increasing by 4.08 times [15]. Meanwhile, increasing symptom burden is associated with higher health care resource utilization with a detrimental impact on work productivity [16]. Therefore, symptomatic treatment has a great clinical appeal for COPD patients. However, treatment according to syndrome difference based on the four diagnostic principles of “diagnosis through observation, diagnosis through auscultation and olfaction, diagnosis through inquiry, and diagnosis through pulse feeling” is the characteristic and one of the advantages of traditional Chinese medicine.

The Yi Qi Gu Biao pill, a compound preparation including Chinese herbs (*Salvia miltiorrhiza *Bunge, Blighted wheat,* Atractylodes macrocephala *Koidz,* Pinellia ternate, Citrus reticulata *Blanco,* Perilla frutescens *(L.) Britt.,* Poria cocos *(Schw.) Wolf,* Saposhnikovia divaricata *(Trucz.) Schischk.,* Coix lacryma-jobi *L. war.ma-yuen (Roman.) Stapf,* Tussilago farfara *L.,* Scutellaria baicalensis *Georgi,* Fritillaria cirrhosa *D. Don, and* Eriobotrya japonica *(Thunb.) Lindl.) is used to treat COPD at a stable stage. We found that the Yi Qi Gu Biao pill improves the quality of life of COPD patients at a stable stage, reducing the number of annual acute exacerbation events [5]. COPD is a publicly recognized heterogeneous disease, with therapeutic responses greatly differing even for patients with the same symptoms and drugs [17]. Based on a previous study, to evaluate the efficacy and safety of the Yi Qi Gu Biao pill for treating the frequent exacerbator phenotype in COPD (lung and spleen qi deficiency syndrome), we conducted a randomized, double-blind, placebo-controlled superiority trial. This study only included the exacerbator phenotype in COPD (lung and spleen qi deficiency syndrome), as subjects to obtain a small sample size in order to standardize the group homogeneity of TCM syndrome differentiation and further clarify the effectiveness or ineffectiveness of the Yi Qi Gu Biao pill.

The Yi Qi Gu Biao pill had little impact on pulmonary function in patients with the exacerbator phenotype in COPD (lung and spleen qi deficiency syndrome), with equivalent efficacy with the Pb during the 3 months of treatment. However, FVC values after two and three months of treatment with Yi Qi Gu Biao pill were increased compared with pretreatment counterparts. Changes of pulmonary function parameters before and after the treatment were not significant. There were no statistically significant differences in the changes of pulmonary function parameters between the two groups after treatment with Yi Qi Gu Biao pill.

Research showed that wheezing correlated with a higher possibility of acute exacerbation. Even among those who were treated according to the GOLD 2011 guidelines, patients with wheezing still had worse symptom scores and more exacerbations. Our study showed that wheezing symptom scores one month after treatment with Yi Qi Gu Biao pill were improved compared with those of the Pb group. Meanwhile, cough, sputum, and wheezing scores after one month of treatment with the Yi Qi Gu Biao pill were improved compared to pretreatment values. However, in the Pb group the cough, sputum, and wheezing scores did not improve until the third month suggesting that Yi Qi Gu Biao pill can cooperate with Western medicine to shorten the duration of cough, sputum, and wheezing symptoms in frequent exacerbator phenotype in COPD.

COPD assessment test (CAT) is a new health-related scale evaluating the health status of COPD patients [18], mainly for COPD patients at a stable stage [19]. After three months of treatment with the Yi Qi Gu Biao pill, CAT scores were reduced compared with the Pb group, indicating that prolonged and sustained administration of the drug is required for its effects of improving syndrome scores and the health status of patients (≥2 months). Interestingly, Yi Qi Gu Biao pill could improve CAT scores after one month, as demonstrated by comparing pretreatment values with those after treatment. However, the degree of improvement varied, as reflected in differences between the two groups before and after the treatment; this means three months of treatment with the Yi Qi Gu Biao pill results in a higher degree of improvement of CAT scores compared with Pb administration.

Improvement of TCM syndrome in the patients was better than Pb group at 1 and 3 months after treatment with the Yi Qi Gu Biao pill. Interestingly, TCM syndrome scores were improved compared with pretreatment values, after one month of treatment with the Yi Qi Gu Biao pill. However, such difference occurred in the Pb group only after two months of treatment.

Substantial placebo effect sizes have been documented for at least 50 years for various psychiatric symptoms [20]. Previous systematic reviews and meta-analyses have found placebo effect sizes that were inconsistent between studies and that have increased over time [21]. Findings [22] indicate that interactions between personality type and environmental cues may contribute to placebo responding. So, placebo effects, including patient's cognition, expectation, attention, preference, and communication with doctor as well as doctor's suggestion, expectation, and indirect regulation of diagnosis and treatment environment on patient's psychology, were essential factors for therapy efficacy. So, in this study, it showed improvements within the placebo groups.

## 5. Conclusion

The Yi Qi Gu Biao pill can improve wheezing, health status, and TCM syndrome in patients with frequent exacerbator phenotype in COPD (lung and spleen qi deficiency syndrome) and also can shorten the durations of cough, sputum, and wheezing.

## Supplementary Material

Figure 1: Pulmonary functions in the two groups after one month of treatment.
Figure 2: Pulmonary functions in the two groups after two months of treatment.
Figure 3: Pulmonary functions in the two groups after three months of treatment.
Figure 4: CAT, mMRC, and TCM syndrome scores in the two groups after one month.
Figure 5: CAT, mMRC, and TCM syndrome scores in the two groups after two months of treatment.
Figure 6: CAT, mMRC, and TCM syndrome scores in the two groups after three months of treatment.
Figure 7: Pulmonary functions in the YQGB group after one month and before treatment.
Figure 8: Pulmonary functions in the YQGB group after two months and before the treatment.
Figure 9: Pulmonary functions in the YQGB group after three months and before the treatment.
Figure 10: Pulmonary functions in the Pb group after one month and before the treatment.
Figure 11: Pulmonary functions in the Pb group after two months and before the treatment.
Figure 12: Pulmonary functions in the Pb group after three months and before the treatment.
Figure 13: CAT and mMRC scores in the YQGB group after one month and before the treatment.
Figure 14: CAT and mMRC in the Pb group after one month and before the treatment.
Figure 15: CAT and mMRC in the YQGB group after two months and before the treatment.
Figure 16: CAT and mMRC in the Pb group after two months and before the treatment.
Figure 17: CAT and mMRC in the YQGB group after three months and before the treatment.
Figure 18: CAT and mMRC in the Pb group after three months and before the treatment.

## Figures and Tables

**Table 1:** Characteristics of the study groups.

	YQGB	Pb
Demographic data	*n *= 33	*n* = 31
Age (years)	68.73 ± 7.16	70.81 ± 5.50
Gender (male/female)	26/7	21/10
BMI	24.04 ± 3.86	25.40 ± 4.43
Height	164.85 ± 6.72	164.61 ± 7.68
Weight	65.55 ± 12.38	68.55 ± 10.82
Smoking habit	*n *= 33	*n* = 31
Smoking	29	21
Non smoking	4	10
Course of disease	*n* = 33	*n *= 31
Course of disease (years)	16.28 ± 11.22	17.60 ± 14.91
Scale score	*n *= 33	*n *= 31
CAT	16.30 ± 5.80	16.81 ± 9.13
mMRC	2.00 ± 0.79	2.23 ± 1.06
Cough	2.85 ± 1.66	2.13 ± 1.71
Sputum	2.18 ± 1.04	1.97 ± 1.56
Wheezing	2.73 ± 1.31	3.23 ± 1.69
TCM symptoms	12.97 ± 3.85	12.48 ± 6.62
Spirometry	*n *= 33	*n *= 28
FEV1/FVC	53.37 ± 10.74	50.87 ± 11.95
FEV1	2.76 ± 7.79	1.14 ± 0.57

**Table 2:** The change of CAT between the YQGB and Pb groups.

	The change of CAT after one month (*n*)		The change of CAT after two months (*n*)		The change of CAT after three months (*n*)	
	≥2 points	<2 points	≥2 points	<2 points	≥2 points	<2 points
YQGB	27	5	32	1	33	0
Pb	18	10	20	4	25	6
*X* ^2^	3.214		3.229		7.048	
*P*	0.073		0.072		0.01	
