# Supplementary figures and images for: A Randomized Controlled Study of the Yi Qi Gu Biao Pill in the Treatment of Frequent Exacerbator Phenotype in Chronic Obstructive Pulmonary Disease (Lung and Spleen Qi Deficiency Syndrome)
Source: Evid Based Complement Alternat Med. 2017 Dec 4;2017:9130804. doi: 10.1155/2017/9130804 (PMC5733762; doi:10.1155/2017/9130804)

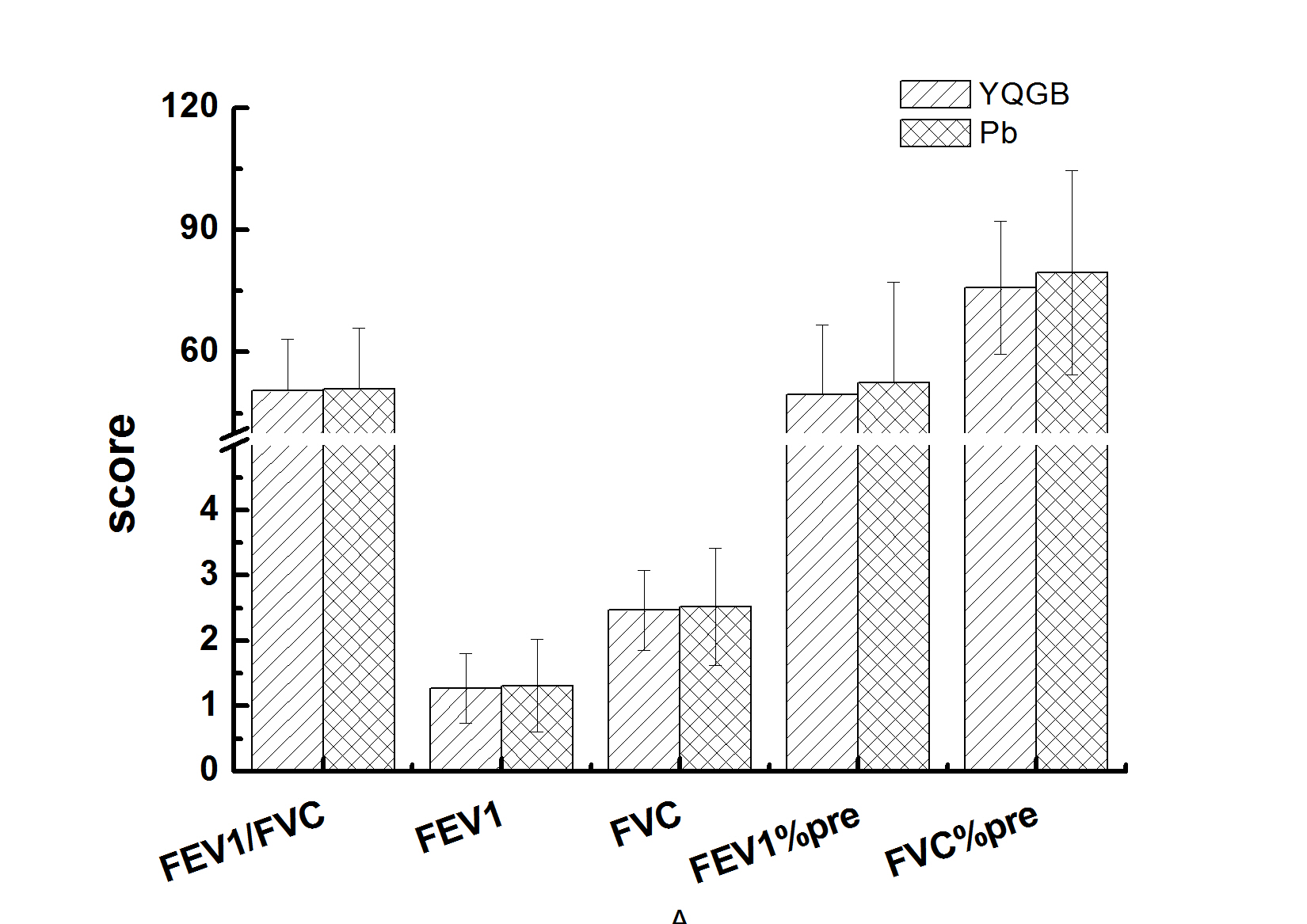

Supplement: Supplementary file 1 — Figure 1: Pulmonary functions in the two groups after one month of treatment. Figure 2: Pulmonary functions in the two groups after two months of treatment. Figure 3: Pulmonary functions in the two groups after three months of treatment. Figure 4: CAT, mMRC, and TCM syndrome scores in the two groups after one month. Figure 5: CAT, mMRC, and TCM syndrome scores in the two groups after two months of treatment. Figure 6: CAT, mMRC, and TCM syndrome scores in the two groups after three months of treatment. Figure 7: Pulmonary functions in the YQGB group after one month and before treatment. Figure 8: Pulmonary functions in the YQGB group after two months and before the treatment. Figure 9: Pulmonary functions in the YQGB group after three months and before the treatment. Figure 10: Pulmonary functions in the Pb group after one month and before the treatment. Figure 11: Pulmonary functions in the Pb group after two months and before the treatment. Figure 12: Pulmonary functions in the Pb group after three months and before the treatment. Figure 13: CAT and mMRC scores in the YQGB group after one month and before the treatment. Figure 14: CAT and mMRC in the Pb group after one month and before the treatment. Figure 15: CAT and mMRC in the YQGB group after two months and before the treatment. Figure 16: CAT and mMRC in the Pb group after two months and before the treatment. Figure 17: CAT and mMRC in the YQGB group after three months and before the treatment. Figure 18: CAT and mMRC in the Pb group after three months and before the treatment. [file 9130804.f1.zip › 图表数据/Figure1.jpg]

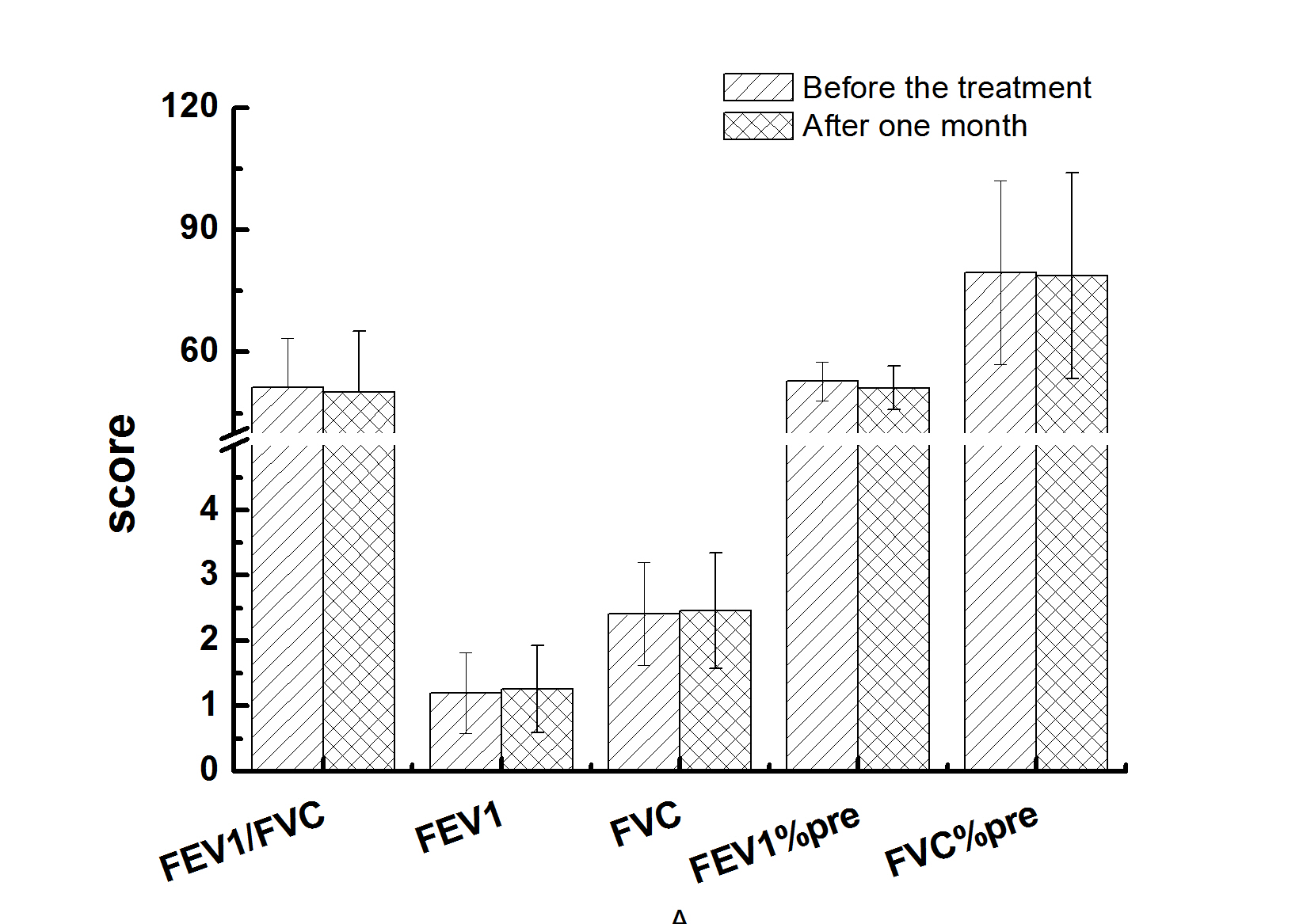

Supplement: Supplementary file 1 — Figure 1: Pulmonary functions in the two groups after one month of treatment. Figure 2: Pulmonary functions in the two groups after two months of treatment. Figure 3: Pulmonary functions in the two groups after three months of treatment. Figure 4: CAT, mMRC, and TCM syndrome scores in the two groups after one month. Figure 5: CAT, mMRC, and TCM syndrome scores in the two groups after two months of treatment. Figure 6: CAT, mMRC, and TCM syndrome scores in the two groups after three months of treatment. Figure 7: Pulmonary functions in the YQGB group after one month and before treatment. Figure 8: Pulmonary functions in the YQGB group after two months and before the treatment. Figure 9: Pulmonary functions in the YQGB group after three months and before the treatment. Figure 10: Pulmonary functions in the Pb group after one month and before the treatment. Figure 11: Pulmonary functions in the Pb group after two months and before the treatment. Figure 12: Pulmonary functions in the Pb group after three months and before the treatment. Figure 13: CAT and mMRC scores in the YQGB group after one month and before the treatment. Figure 14: CAT and mMRC in the Pb group after one month and before the treatment. Figure 15: CAT and mMRC in the YQGB group after two months and before the treatment. Figure 16: CAT and mMRC in the Pb group after two months and before the treatment. Figure 17: CAT and mMRC in the YQGB group after three months and before the treatment. Figure 18: CAT and mMRC in the Pb group after three months and before the treatment. [file 9130804.f1.zip › 图表数据/Figure10.jpg]

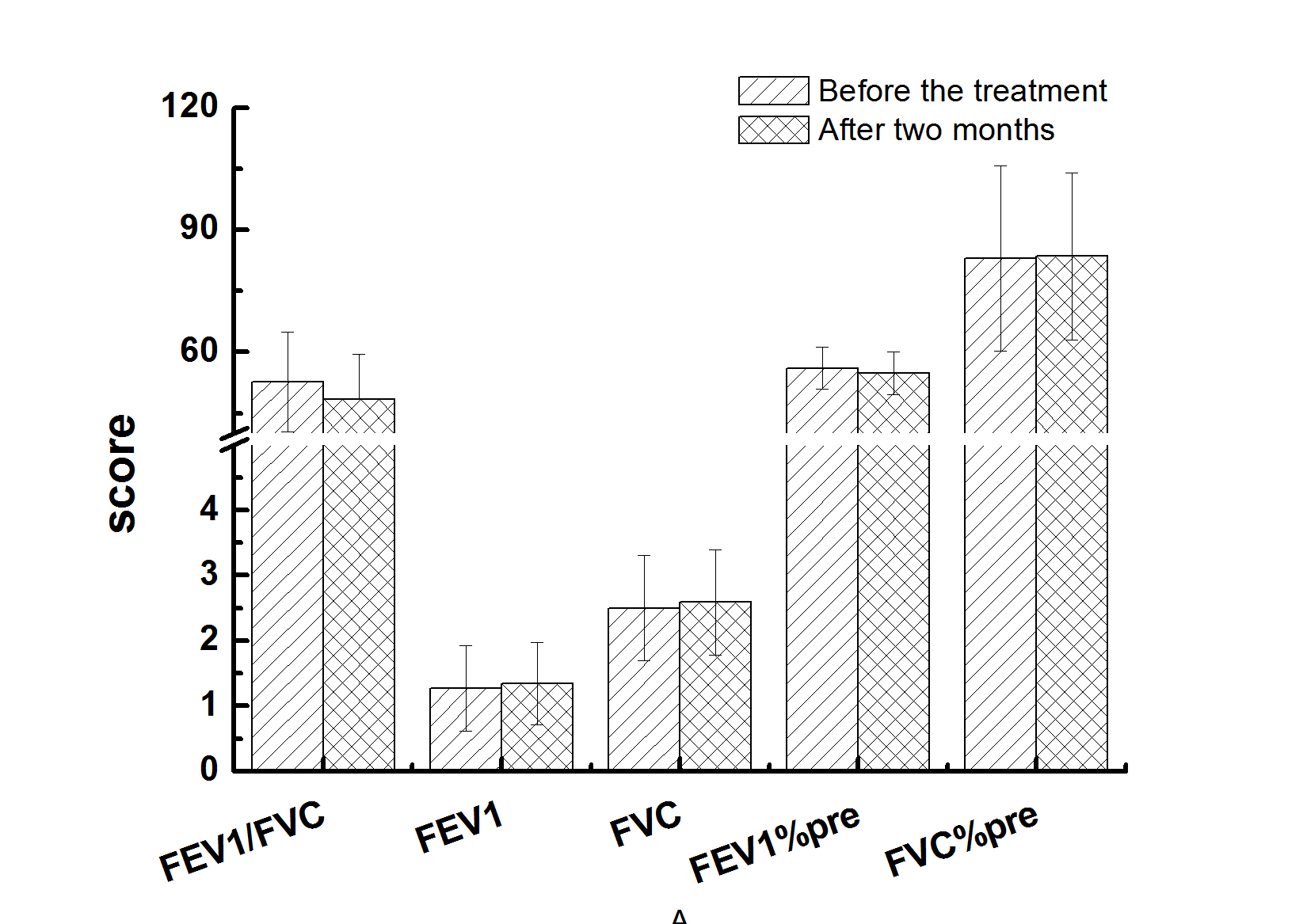

Supplement: Supplementary file 1 — Figure 1: Pulmonary functions in the two groups after one month of treatment. Figure 2: Pulmonary functions in the two groups after two months of treatment. Figure 3: Pulmonary functions in the two groups after three months of treatment. Figure 4: CAT, mMRC, and TCM syndrome scores in the two groups after one month. Figure 5: CAT, mMRC, and TCM syndrome scores in the two groups after two months of treatment. Figure 6: CAT, mMRC, and TCM syndrome scores in the two groups after three months of treatment. Figure 7: Pulmonary functions in the YQGB group after one month and before treatment. Figure 8: Pulmonary functions in the YQGB group after two months and before the treatment. Figure 9: Pulmonary functions in the YQGB group after three months and before the treatment. Figure 10: Pulmonary functions in the Pb group after one month and before the treatment. Figure 11: Pulmonary functions in the Pb group after two months and before the treatment. Figure 12: Pulmonary functions in the Pb group after three months and before the treatment. Figure 13: CAT and mMRC scores in the YQGB group after one month and before the treatment. Figure 14: CAT and mMRC in the Pb group after one month and before the treatment. Figure 15: CAT and mMRC in the YQGB group after two months and before the treatment. Figure 16: CAT and mMRC in the Pb group after two months and before the treatment. Figure 17: CAT and mMRC in the YQGB group after three months and before the treatment. Figure 18: CAT and mMRC in the Pb group after three months and before the treatment. [file 9130804.f1.zip › 图表数据/Figure11.jpg]

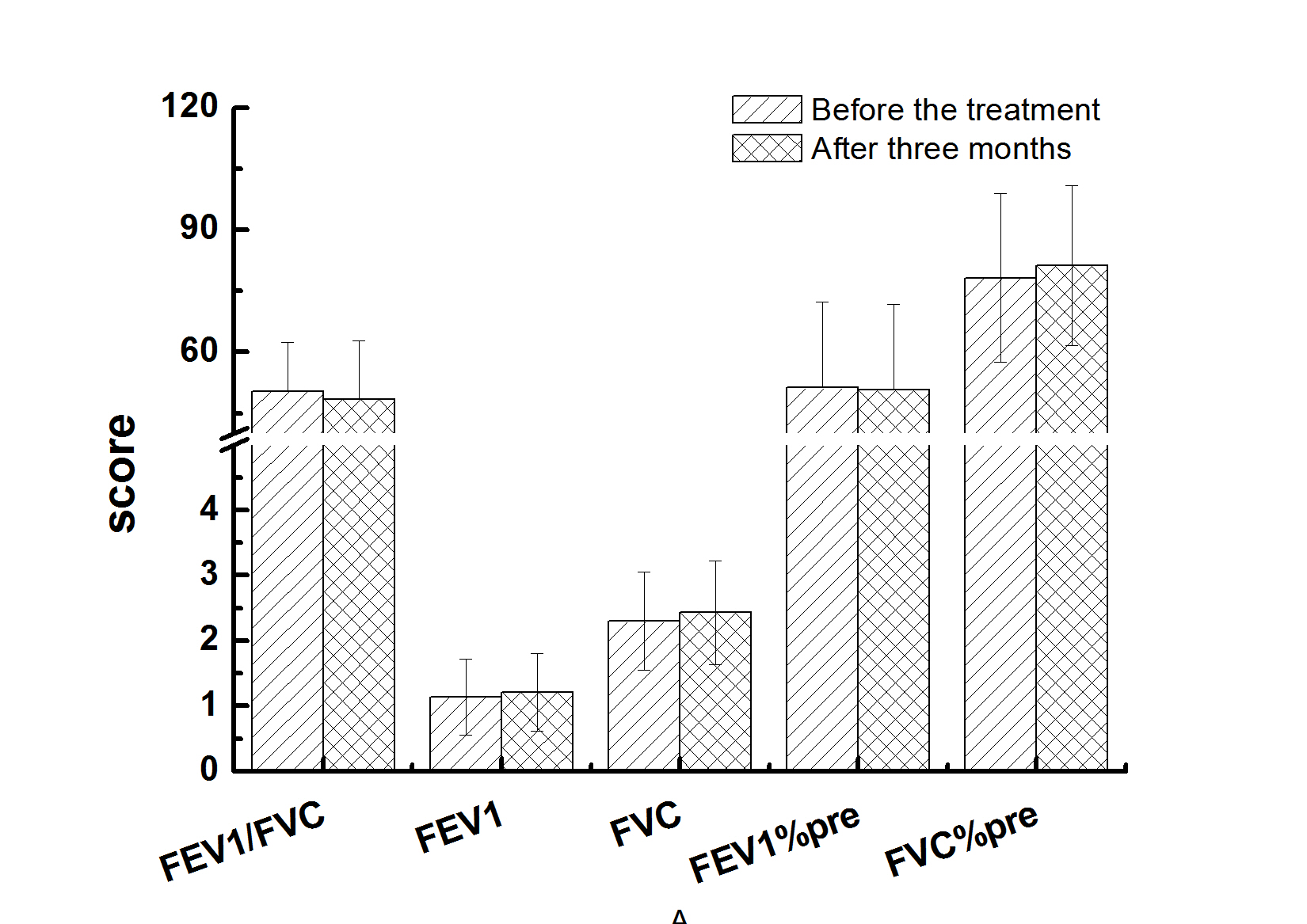

Supplement: Supplementary file 1 — Figure 1: Pulmonary functions in the two groups after one month of treatment. Figure 2: Pulmonary functions in the two groups after two months of treatment. Figure 3: Pulmonary functions in the two groups after three months of treatment. Figure 4: CAT, mMRC, and TCM syndrome scores in the two groups after one month. Figure 5: CAT, mMRC, and TCM syndrome scores in the two groups after two months of treatment. Figure 6: CAT, mMRC, and TCM syndrome scores in the two groups after three months of treatment. Figure 7: Pulmonary functions in the YQGB group after one month and before treatment. Figure 8: Pulmonary functions in the YQGB group after two months and before the treatment. Figure 9: Pulmonary functions in the YQGB group after three months and before the treatment. Figure 10: Pulmonary functions in the Pb group after one month and before the treatment. Figure 11: Pulmonary functions in the Pb group after two months and before the treatment. Figure 12: Pulmonary functions in the Pb group after three months and before the treatment. Figure 13: CAT and mMRC scores in the YQGB group after one month and before the treatment. Figure 14: CAT and mMRC in the Pb group after one month and before the treatment. Figure 15: CAT and mMRC in the YQGB group after two months and before the treatment. Figure 16: CAT and mMRC in the Pb group after two months and before the treatment. Figure 17: CAT and mMRC in the YQGB group after three months and before the treatment. Figure 18: CAT and mMRC in the Pb group after three months and before the treatment. [file 9130804.f1.zip › 图表数据/Figure12.jpg]

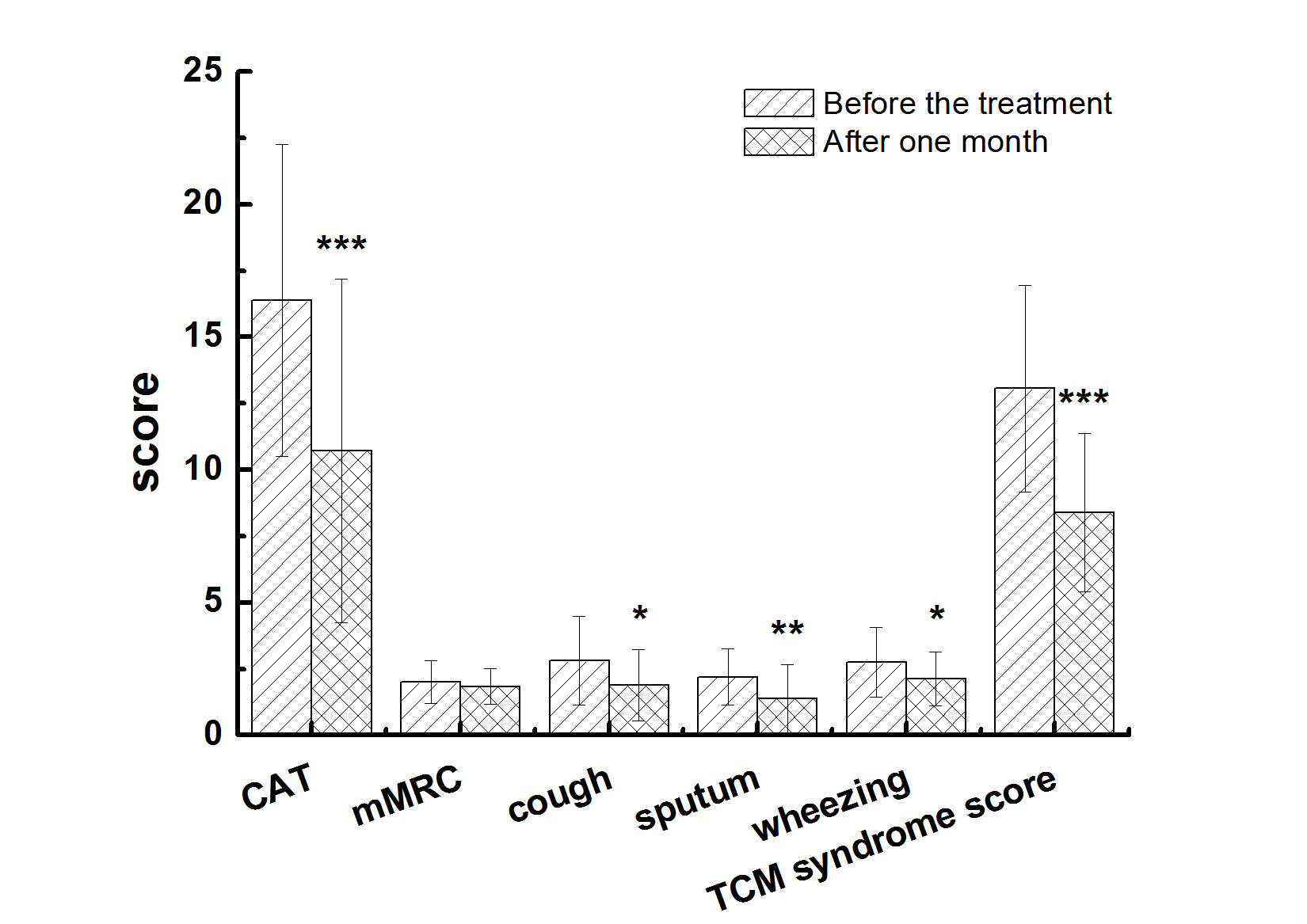

Supplement: Supplementary file 1 — Figure 1: Pulmonary functions in the two groups after one month of treatment. Figure 2: Pulmonary functions in the two groups after two months of treatment. Figure 3: Pulmonary functions in the two groups after three months of treatment. Figure 4: CAT, mMRC, and TCM syndrome scores in the two groups after one month. Figure 5: CAT, mMRC, and TCM syndrome scores in the two groups after two months of treatment. Figure 6: CAT, mMRC, and TCM syndrome scores in the two groups after three months of treatment. Figure 7: Pulmonary functions in the YQGB group after one month and before treatment. Figure 8: Pulmonary functions in the YQGB group after two months and before the treatment. Figure 9: Pulmonary functions in the YQGB group after three months and before the treatment. Figure 10: Pulmonary functions in the Pb group after one month and before the treatment. Figure 11: Pulmonary functions in the Pb group after two months and before the treatment. Figure 12: Pulmonary functions in the Pb group after three months and before the treatment. Figure 13: CAT and mMRC scores in the YQGB group after one month and before the treatment. Figure 14: CAT and mMRC in the Pb group after one month and before the treatment. Figure 15: CAT and mMRC in the YQGB group after two months and before the treatment. Figure 16: CAT and mMRC in the Pb group after two months and before the treatment. Figure 17: CAT and mMRC in the YQGB group after three months and before the treatment. Figure 18: CAT and mMRC in the Pb group after three months and before the treatment. [file 9130804.f1.zip › 图表数据/Figure13.jpg]

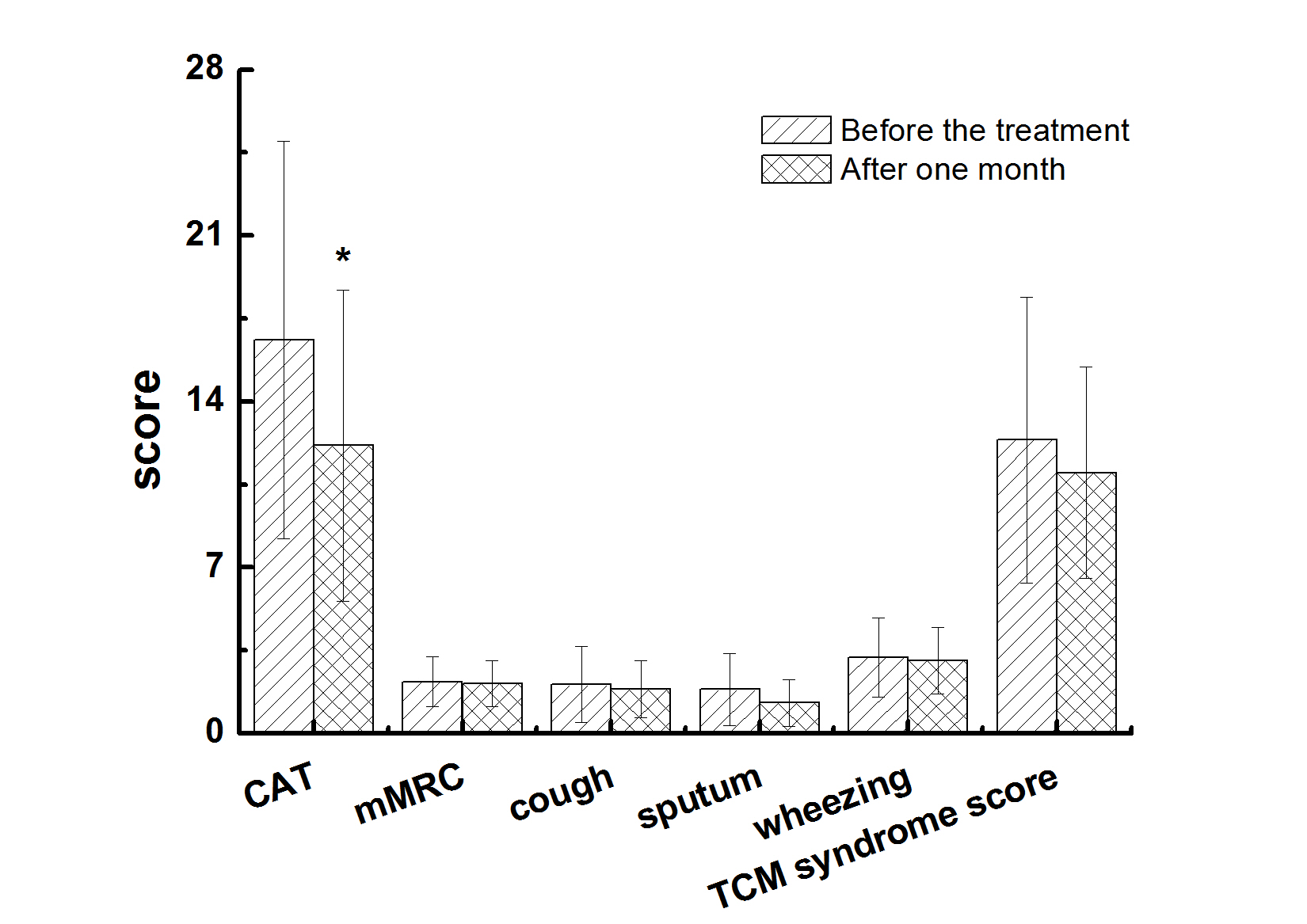

Supplement: Supplementary file 1 — Figure 1: Pulmonary functions in the two groups after one month of treatment. Figure 2: Pulmonary functions in the two groups after two months of treatment. Figure 3: Pulmonary functions in the two groups after three months of treatment. Figure 4: CAT, mMRC, and TCM syndrome scores in the two groups after one month. Figure 5: CAT, mMRC, and TCM syndrome scores in the two groups after two months of treatment. Figure 6: CAT, mMRC, and TCM syndrome scores in the two groups after three months of treatment. Figure 7: Pulmonary functions in the YQGB group after one month and before treatment. Figure 8: Pulmonary functions in the YQGB group after two months and before the treatment. Figure 9: Pulmonary functions in the YQGB group after three months and before the treatment. Figure 10: Pulmonary functions in the Pb group after one month and before the treatment. Figure 11: Pulmonary functions in the Pb group after two months and before the treatment. Figure 12: Pulmonary functions in the Pb group after three months and before the treatment. Figure 13: CAT and mMRC scores in the YQGB group after one month and before the treatment. Figure 14: CAT and mMRC in the Pb group after one month and before the treatment. Figure 15: CAT and mMRC in the YQGB group after two months and before the treatment. Figure 16: CAT and mMRC in the Pb group after two months and before the treatment. Figure 17: CAT and mMRC in the YQGB group after three months and before the treatment. Figure 18: CAT and mMRC in the Pb group after three months and before the treatment. [file 9130804.f1.zip › 图表数据/Figure14.jpg]

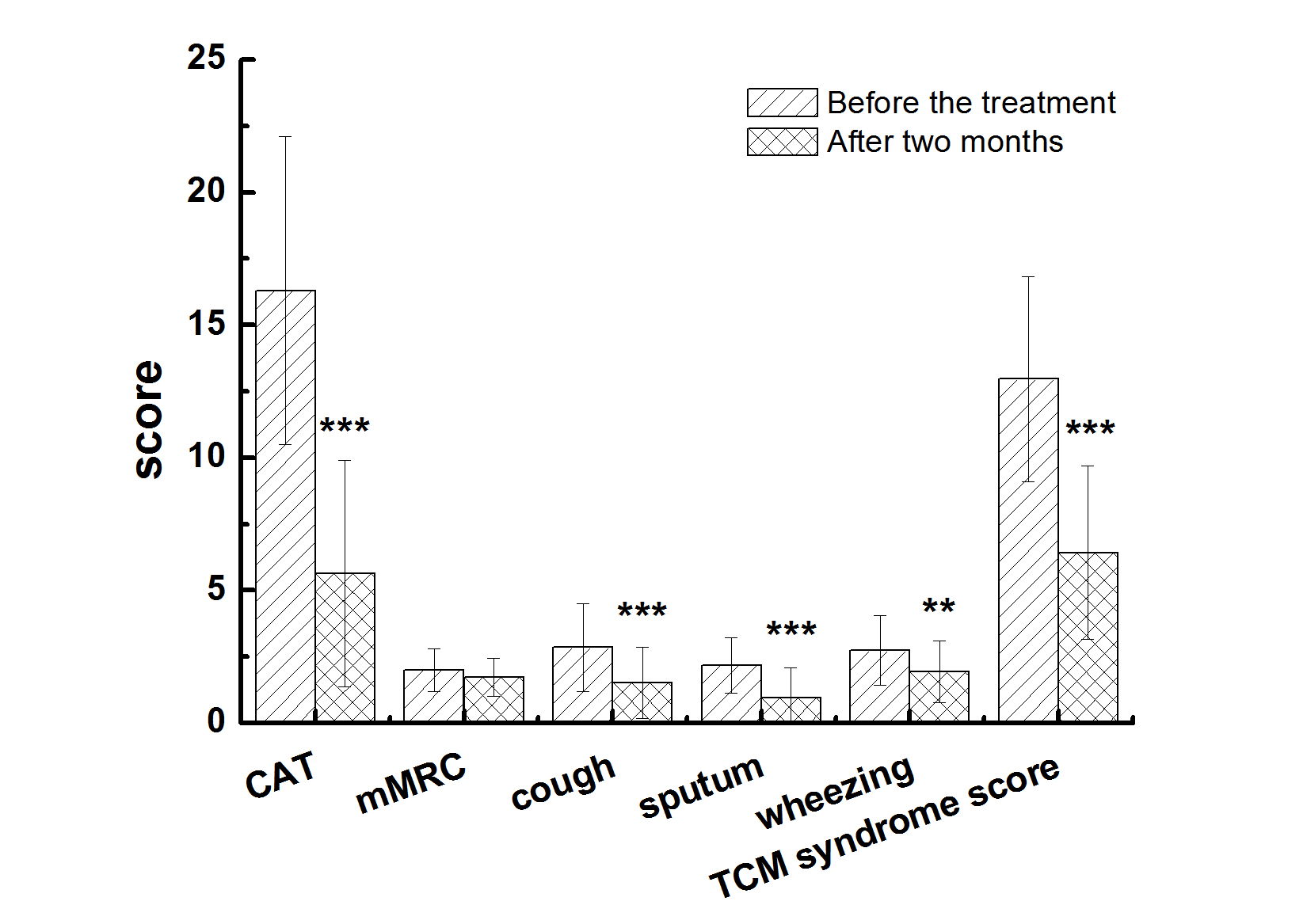

Supplement: Supplementary file 1 — Figure 1: Pulmonary functions in the two groups after one month of treatment. Figure 2: Pulmonary functions in the two groups after two months of treatment. Figure 3: Pulmonary functions in the two groups after three months of treatment. Figure 4: CAT, mMRC, and TCM syndrome scores in the two groups after one month. Figure 5: CAT, mMRC, and TCM syndrome scores in the two groups after two months of treatment. Figure 6: CAT, mMRC, and TCM syndrome scores in the two groups after three months of treatment. Figure 7: Pulmonary functions in the YQGB group after one month and before treatment. Figure 8: Pulmonary functions in the YQGB group after two months and before the treatment. Figure 9: Pulmonary functions in the YQGB group after three months and before the treatment. Figure 10: Pulmonary functions in the Pb group after one month and before the treatment. Figure 11: Pulmonary functions in the Pb group after two months and before the treatment. Figure 12: Pulmonary functions in the Pb group after three months and before the treatment. Figure 13: CAT and mMRC scores in the YQGB group after one month and before the treatment. Figure 14: CAT and mMRC in the Pb group after one month and before the treatment. Figure 15: CAT and mMRC in the YQGB group after two months and before the treatment. Figure 16: CAT and mMRC in the Pb group after two months and before the treatment. Figure 17: CAT and mMRC in the YQGB group after three months and before the treatment. Figure 18: CAT and mMRC in the Pb group after three months and before the treatment. [file 9130804.f1.zip › 图表数据/Figure15.jpg]

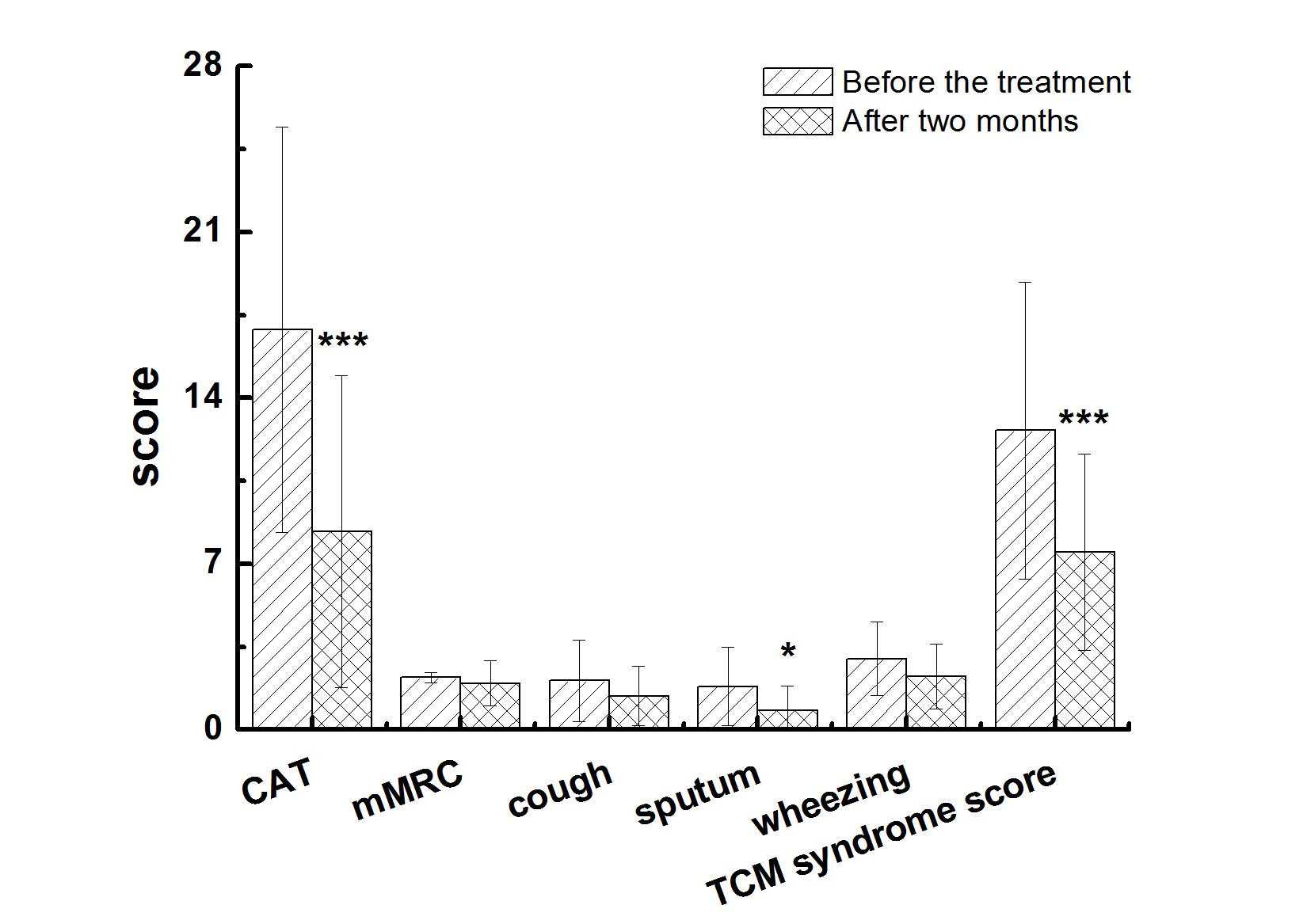

Supplement: Supplementary file 1 — Figure 1: Pulmonary functions in the two groups after one month of treatment. Figure 2: Pulmonary functions in the two groups after two months of treatment. Figure 3: Pulmonary functions in the two groups after three months of treatment. Figure 4: CAT, mMRC, and TCM syndrome scores in the two groups after one month. Figure 5: CAT, mMRC, and TCM syndrome scores in the two groups after two months of treatment. Figure 6: CAT, mMRC, and TCM syndrome scores in the two groups after three months of treatment. Figure 7: Pulmonary functions in the YQGB group after one month and before treatment. Figure 8: Pulmonary functions in the YQGB group after two months and before the treatment. Figure 9: Pulmonary functions in the YQGB group after three months and before the treatment. Figure 10: Pulmonary functions in the Pb group after one month and before the treatment. Figure 11: Pulmonary functions in the Pb group after two months and before the treatment. Figure 12: Pulmonary functions in the Pb group after three months and before the treatment. Figure 13: CAT and mMRC scores in the YQGB group after one month and before the treatment. Figure 14: CAT and mMRC in the Pb group after one month and before the treatment. Figure 15: CAT and mMRC in the YQGB group after two months and before the treatment. Figure 16: CAT and mMRC in the Pb group after two months and before the treatment. Figure 17: CAT and mMRC in the YQGB group after three months and before the treatment. Figure 18: CAT and mMRC in the Pb group after three months and before the treatment. [file 9130804.f1.zip › 图表数据/Figure16.jpg]

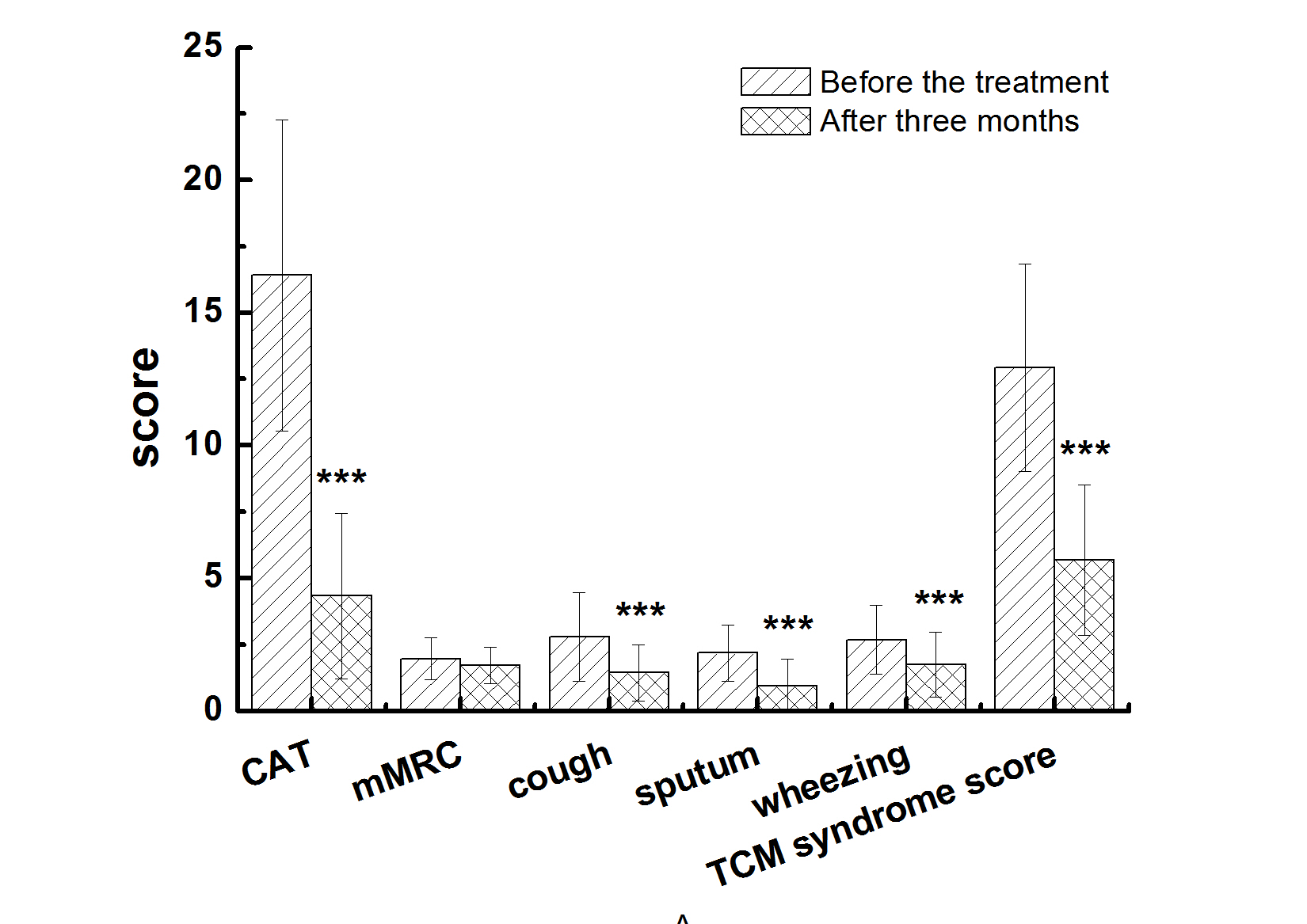

Supplement: Supplementary file 1 — Figure 1: Pulmonary functions in the two groups after one month of treatment. Figure 2: Pulmonary functions in the two groups after two months of treatment. Figure 3: Pulmonary functions in the two groups after three months of treatment. Figure 4: CAT, mMRC, and TCM syndrome scores in the two groups after one month. Figure 5: CAT, mMRC, and TCM syndrome scores in the two groups after two months of treatment. Figure 6: CAT, mMRC, and TCM syndrome scores in the two groups after three months of treatment. Figure 7: Pulmonary functions in the YQGB group after one month and before treatment. Figure 8: Pulmonary functions in the YQGB group after two months and before the treatment. Figure 9: Pulmonary functions in the YQGB group after three months and before the treatment. Figure 10: Pulmonary functions in the Pb group after one month and before the treatment. Figure 11: Pulmonary functions in the Pb group after two months and before the treatment. Figure 12: Pulmonary functions in the Pb group after three months and before the treatment. Figure 13: CAT and mMRC scores in the YQGB group after one month and before the treatment. Figure 14: CAT and mMRC in the Pb group after one month and before the treatment. Figure 15: CAT and mMRC in the YQGB group after two months and before the treatment. Figure 16: CAT and mMRC in the Pb group after two months and before the treatment. Figure 17: CAT and mMRC in the YQGB group after three months and before the treatment. Figure 18: CAT and mMRC in the Pb group after three months and before the treatment. [file 9130804.f1.zip › 图表数据/Figure17.jpg]

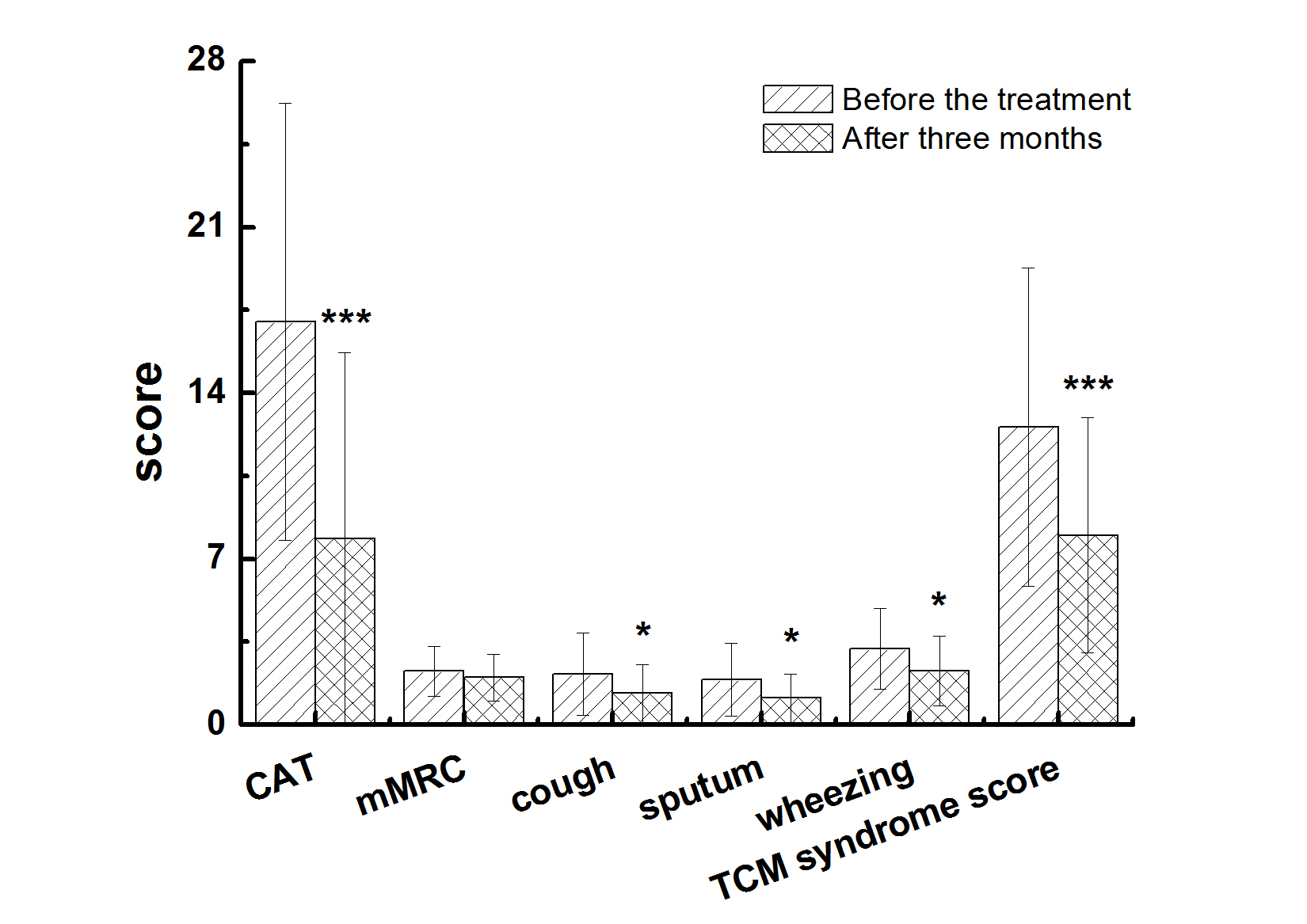

Supplement: Supplementary file 1 — Figure 1: Pulmonary functions in the two groups after one month of treatment. Figure 2: Pulmonary functions in the two groups after two months of treatment. Figure 3: Pulmonary functions in the two groups after three months of treatment. Figure 4: CAT, mMRC, and TCM syndrome scores in the two groups after one month. Figure 5: CAT, mMRC, and TCM syndrome scores in the two groups after two months of treatment. Figure 6: CAT, mMRC, and TCM syndrome scores in the two groups after three months of treatment. Figure 7: Pulmonary functions in the YQGB group after one month and before treatment. Figure 8: Pulmonary functions in the YQGB group after two months and before the treatment. Figure 9: Pulmonary functions in the YQGB group after three months and before the treatment. Figure 10: Pulmonary functions in the Pb group after one month and before the treatment. Figure 11: Pulmonary functions in the Pb group after two months and before the treatment. Figure 12: Pulmonary functions in the Pb group after three months and before the treatment. Figure 13: CAT and mMRC scores in the YQGB group after one month and before the treatment. Figure 14: CAT and mMRC in the Pb group after one month and before the treatment. Figure 15: CAT and mMRC in the YQGB group after two months and before the treatment. Figure 16: CAT and mMRC in the Pb group after two months and before the treatment. Figure 17: CAT and mMRC in the YQGB group after three months and before the treatment. Figure 18: CAT and mMRC in the Pb group after three months and before the treatment. [file 9130804.f1.zip › 图表数据/Figure18.jpg]

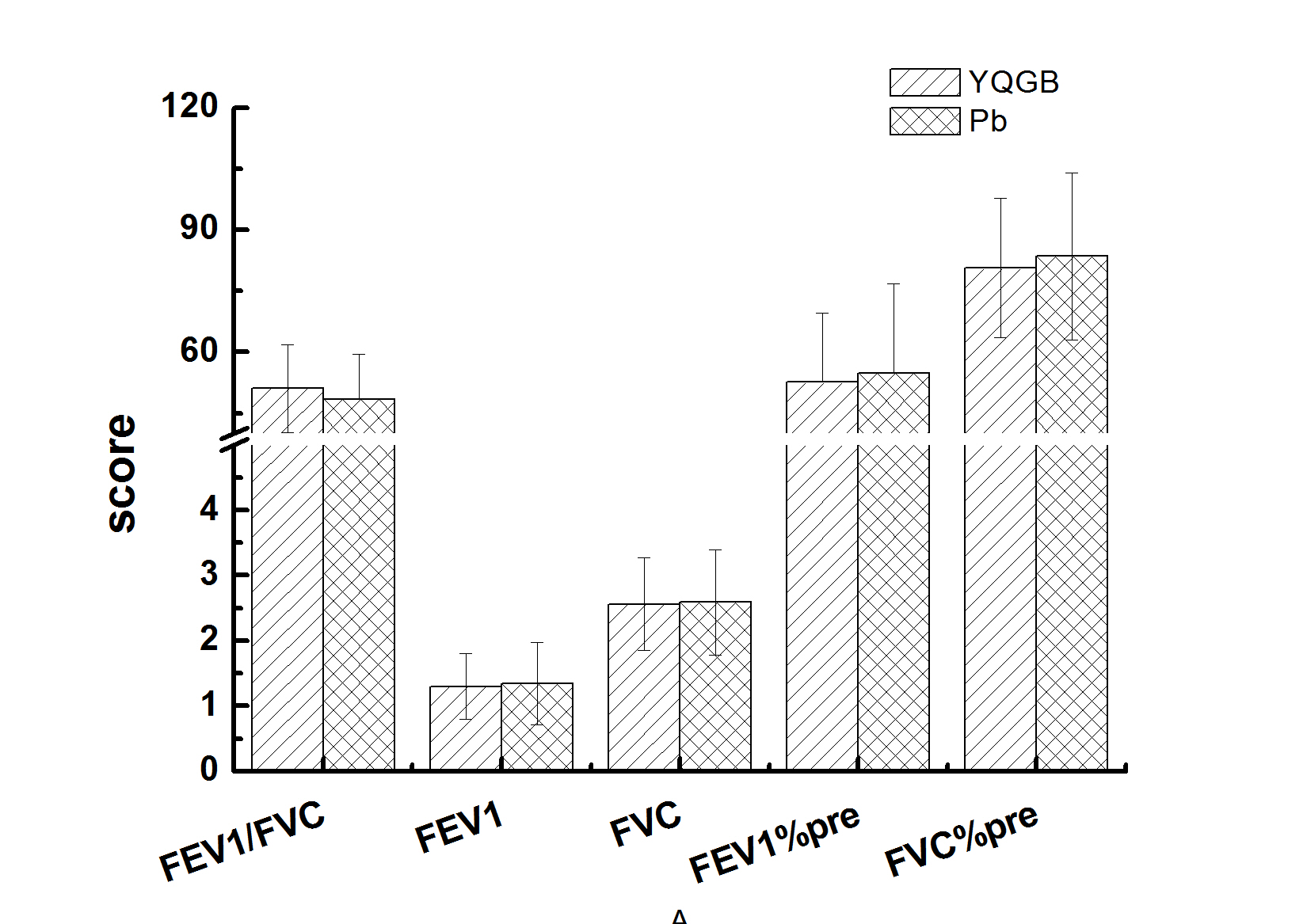

Supplement: Supplementary file 1 — Figure 1: Pulmonary functions in the two groups after one month of treatment. Figure 2: Pulmonary functions in the two groups after two months of treatment. Figure 3: Pulmonary functions in the two groups after three months of treatment. Figure 4: CAT, mMRC, and TCM syndrome scores in the two groups after one month. Figure 5: CAT, mMRC, and TCM syndrome scores in the two groups after two months of treatment. Figure 6: CAT, mMRC, and TCM syndrome scores in the two groups after three months of treatment. Figure 7: Pulmonary functions in the YQGB group after one month and before treatment. Figure 8: Pulmonary functions in the YQGB group after two months and before the treatment. Figure 9: Pulmonary functions in the YQGB group after three months and before the treatment. Figure 10: Pulmonary functions in the Pb group after one month and before the treatment. Figure 11: Pulmonary functions in the Pb group after two months and before the treatment. Figure 12: Pulmonary functions in the Pb group after three months and before the treatment. Figure 13: CAT and mMRC scores in the YQGB group after one month and before the treatment. Figure 14: CAT and mMRC in the Pb group after one month and before the treatment. Figure 15: CAT and mMRC in the YQGB group after two months and before the treatment. Figure 16: CAT and mMRC in the Pb group after two months and before the treatment. Figure 17: CAT and mMRC in the YQGB group after three months and before the treatment. Figure 18: CAT and mMRC in the Pb group after three months and before the treatment. [file 9130804.f1.zip › 图表数据/Figure2.jpg]

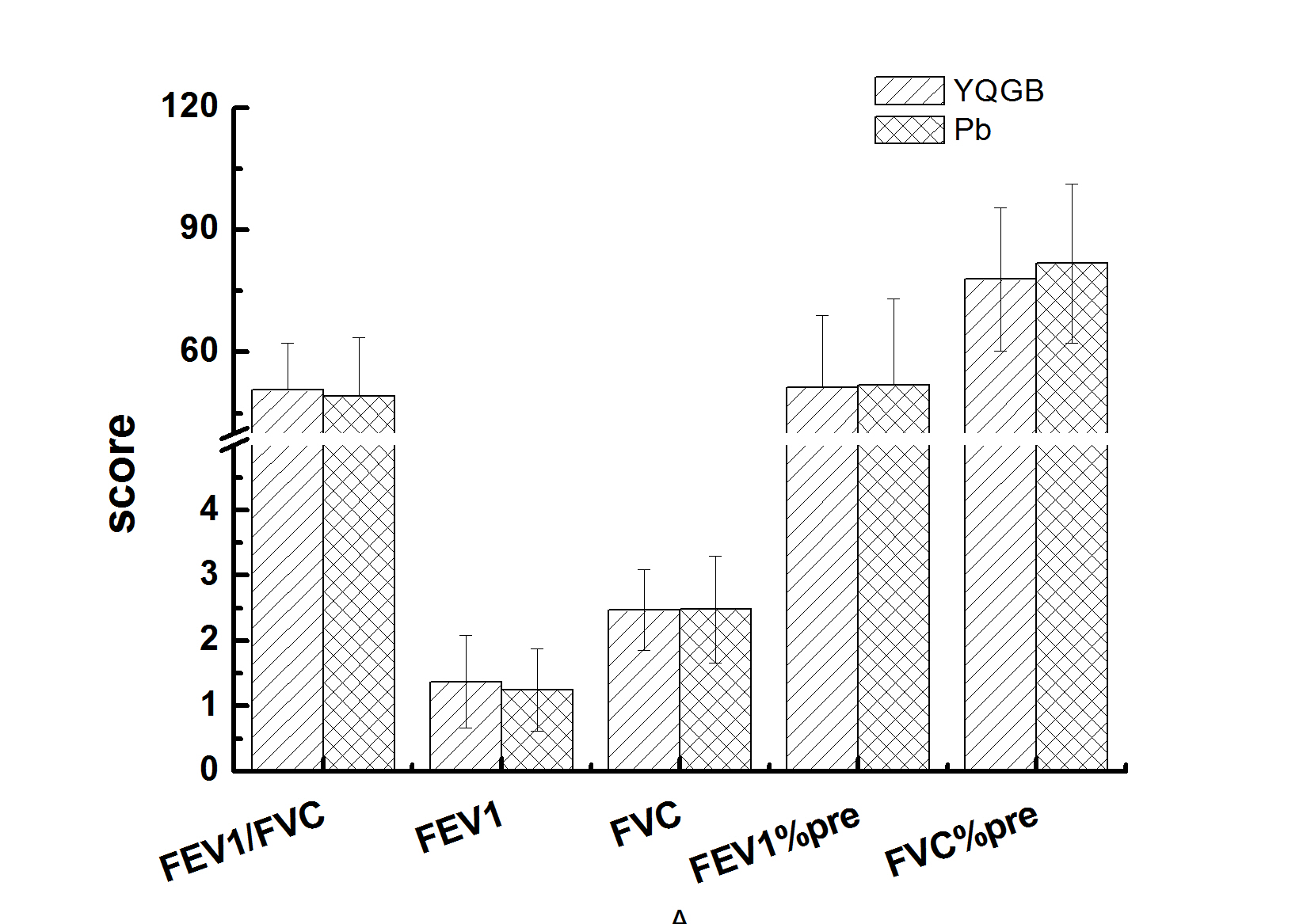

Supplement: Supplementary file 1 — Figure 1: Pulmonary functions in the two groups after one month of treatment. Figure 2: Pulmonary functions in the two groups after two months of treatment. Figure 3: Pulmonary functions in the two groups after three months of treatment. Figure 4: CAT, mMRC, and TCM syndrome scores in the two groups after one month. Figure 5: CAT, mMRC, and TCM syndrome scores in the two groups after two months of treatment. Figure 6: CAT, mMRC, and TCM syndrome scores in the two groups after three months of treatment. Figure 7: Pulmonary functions in the YQGB group after one month and before treatment. Figure 8: Pulmonary functions in the YQGB group after two months and before the treatment. Figure 9: Pulmonary functions in the YQGB group after three months and before the treatment. Figure 10: Pulmonary functions in the Pb group after one month and before the treatment. Figure 11: Pulmonary functions in the Pb group after two months and before the treatment. Figure 12: Pulmonary functions in the Pb group after three months and before the treatment. Figure 13: CAT and mMRC scores in the YQGB group after one month and before the treatment. Figure 14: CAT and mMRC in the Pb group after one month and before the treatment. Figure 15: CAT and mMRC in the YQGB group after two months and before the treatment. Figure 16: CAT and mMRC in the Pb group after two months and before the treatment. Figure 17: CAT and mMRC in the YQGB group after three months and before the treatment. Figure 18: CAT and mMRC in the Pb group after three months and before the treatment. [file 9130804.f1.zip › 图表数据/Figure3.jpg]

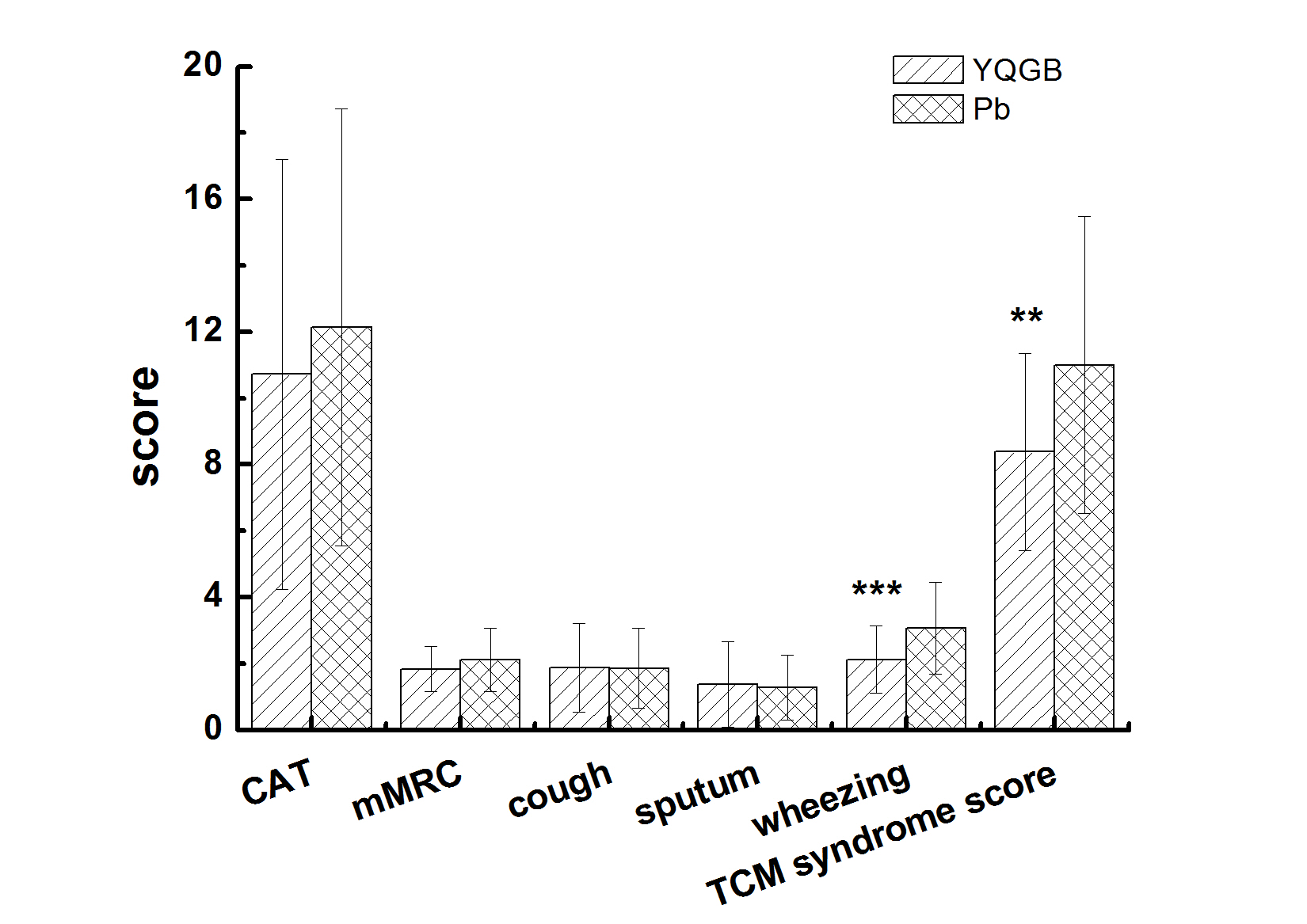

Supplement: Supplementary file 1 — Figure 1: Pulmonary functions in the two groups after one month of treatment. Figure 2: Pulmonary functions in the two groups after two months of treatment. Figure 3: Pulmonary functions in the two groups after three months of treatment. Figure 4: CAT, mMRC, and TCM syndrome scores in the two groups after one month. Figure 5: CAT, mMRC, and TCM syndrome scores in the two groups after two months of treatment. Figure 6: CAT, mMRC, and TCM syndrome scores in the two groups after three months of treatment. Figure 7: Pulmonary functions in the YQGB group after one month and before treatment. Figure 8: Pulmonary functions in the YQGB group after two months and before the treatment. Figure 9: Pulmonary functions in the YQGB group after three months and before the treatment. Figure 10: Pulmonary functions in the Pb group after one month and before the treatment. Figure 11: Pulmonary functions in the Pb group after two months and before the treatment. Figure 12: Pulmonary functions in the Pb group after three months and before the treatment. Figure 13: CAT and mMRC scores in the YQGB group after one month and before the treatment. Figure 14: CAT and mMRC in the Pb group after one month and before the treatment. Figure 15: CAT and mMRC in the YQGB group after two months and before the treatment. Figure 16: CAT and mMRC in the Pb group after two months and before the treatment. Figure 17: CAT and mMRC in the YQGB group after three months and before the treatment. Figure 18: CAT and mMRC in the Pb group after three months and before the treatment. [file 9130804.f1.zip › 图表数据/Figure4.jpg]

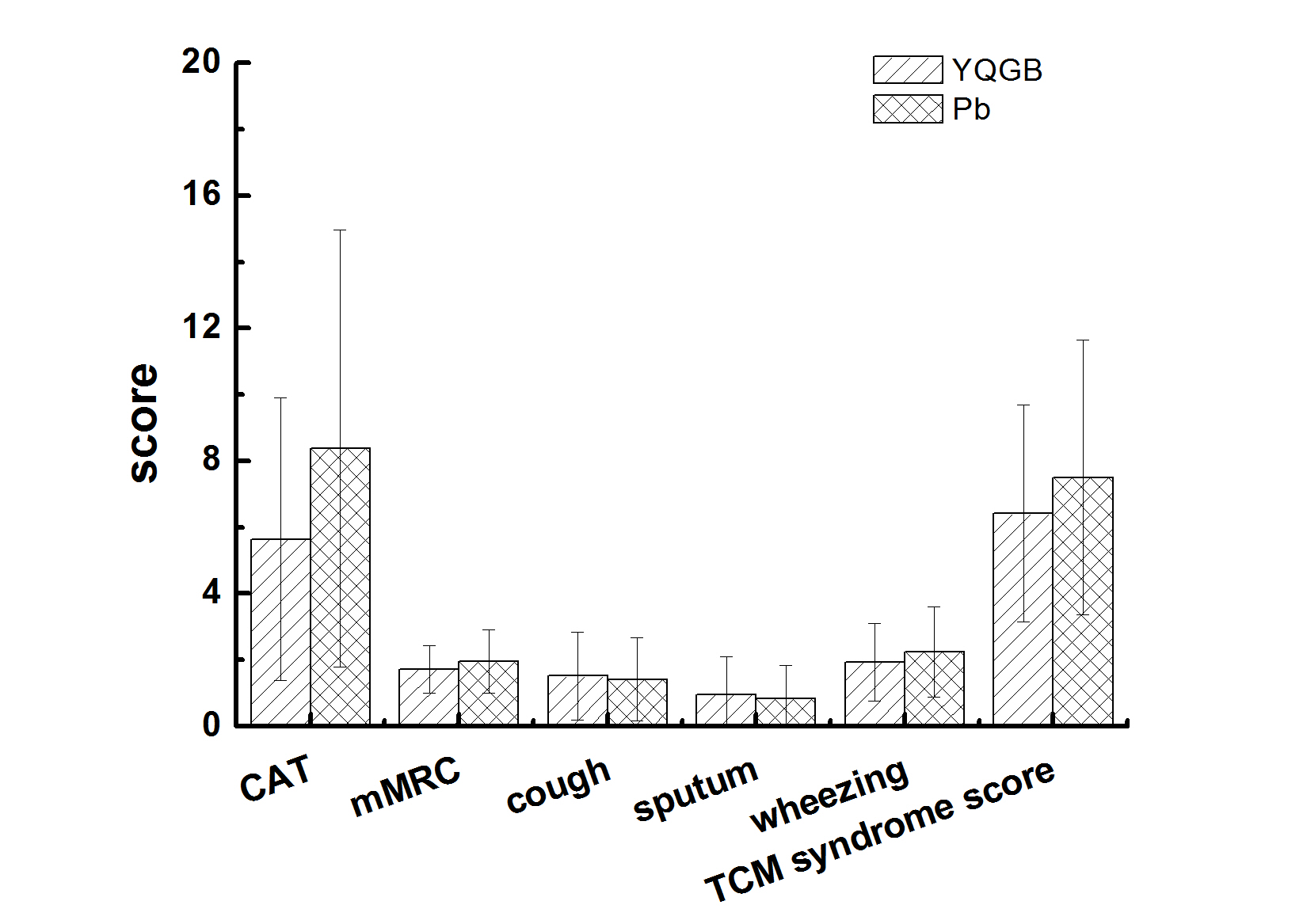

Supplement: Supplementary file 1 — Figure 1: Pulmonary functions in the two groups after one month of treatment. Figure 2: Pulmonary functions in the two groups after two months of treatment. Figure 3: Pulmonary functions in the two groups after three months of treatment. Figure 4: CAT, mMRC, and TCM syndrome scores in the two groups after one month. Figure 5: CAT, mMRC, and TCM syndrome scores in the two groups after two months of treatment. Figure 6: CAT, mMRC, and TCM syndrome scores in the two groups after three months of treatment. Figure 7: Pulmonary functions in the YQGB group after one month and before treatment. Figure 8: Pulmonary functions in the YQGB group after two months and before the treatment. Figure 9: Pulmonary functions in the YQGB group after three months and before the treatment. Figure 10: Pulmonary functions in the Pb group after one month and before the treatment. Figure 11: Pulmonary functions in the Pb group after two months and before the treatment. Figure 12: Pulmonary functions in the Pb group after three months and before the treatment. Figure 13: CAT and mMRC scores in the YQGB group after one month and before the treatment. Figure 14: CAT and mMRC in the Pb group after one month and before the treatment. Figure 15: CAT and mMRC in the YQGB group after two months and before the treatment. Figure 16: CAT and mMRC in the Pb group after two months and before the treatment. Figure 17: CAT and mMRC in the YQGB group after three months and before the treatment. Figure 18: CAT and mMRC in the Pb group after three months and before the treatment. [file 9130804.f1.zip › 图表数据/Figure5.jpg]

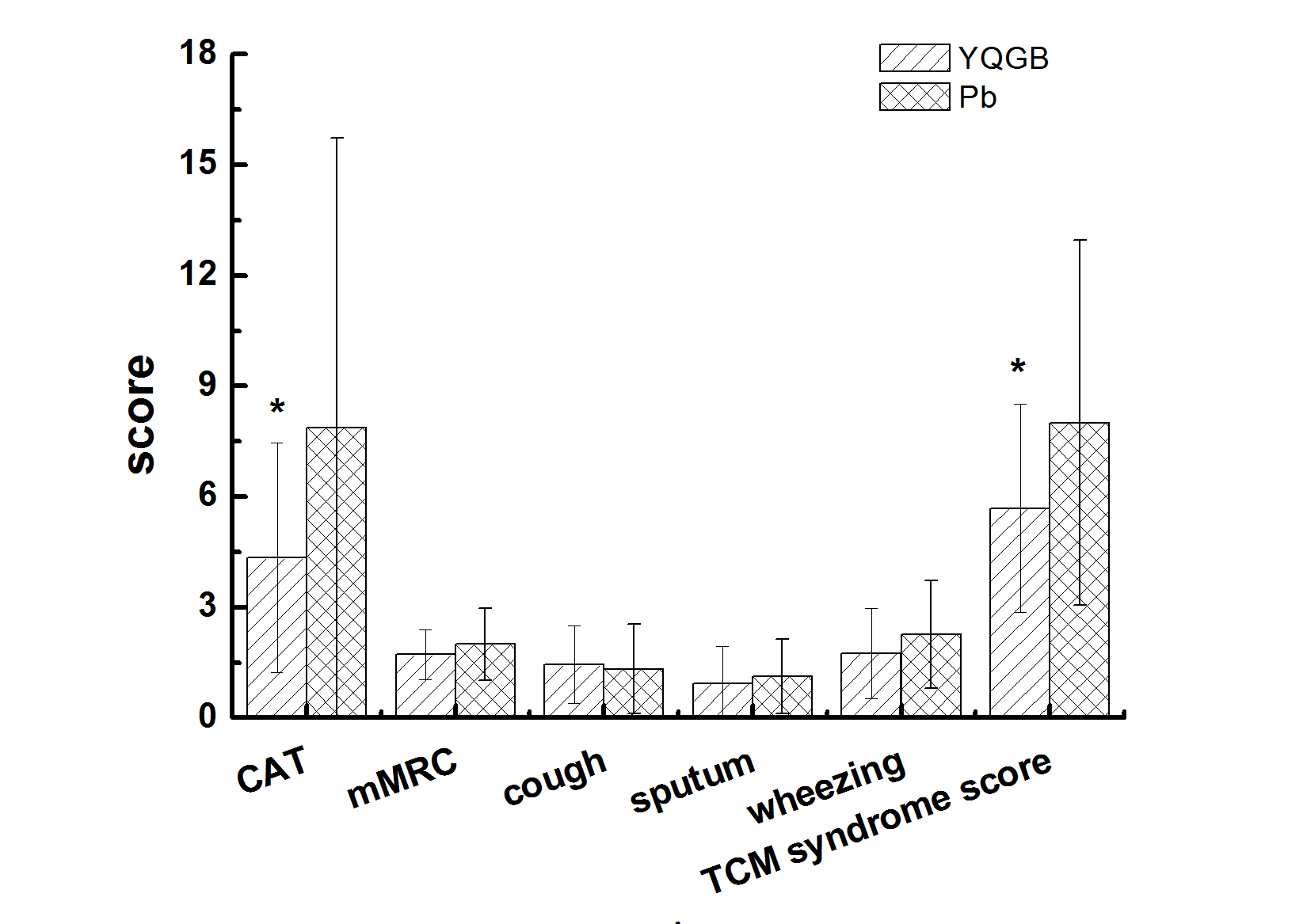

Supplement: Supplementary file 1 — Figure 1: Pulmonary functions in the two groups after one month of treatment. Figure 2: Pulmonary functions in the two groups after two months of treatment. Figure 3: Pulmonary functions in the two groups after three months of treatment. Figure 4: CAT, mMRC, and TCM syndrome scores in the two groups after one month. Figure 5: CAT, mMRC, and TCM syndrome scores in the two groups after two months of treatment. Figure 6: CAT, mMRC, and TCM syndrome scores in the two groups after three months of treatment. Figure 7: Pulmonary functions in the YQGB group after one month and before treatment. Figure 8: Pulmonary functions in the YQGB group after two months and before the treatment. Figure 9: Pulmonary functions in the YQGB group after three months and before the treatment. Figure 10: Pulmonary functions in the Pb group after one month and before the treatment. Figure 11: Pulmonary functions in the Pb group after two months and before the treatment. Figure 12: Pulmonary functions in the Pb group after three months and before the treatment. Figure 13: CAT and mMRC scores in the YQGB group after one month and before the treatment. Figure 14: CAT and mMRC in the Pb group after one month and before the treatment. Figure 15: CAT and mMRC in the YQGB group after two months and before the treatment. Figure 16: CAT and mMRC in the Pb group after two months and before the treatment. Figure 17: CAT and mMRC in the YQGB group after three months and before the treatment. Figure 18: CAT and mMRC in the Pb group after three months and before the treatment. [file 9130804.f1.zip › 图表数据/Figure6.jpg]

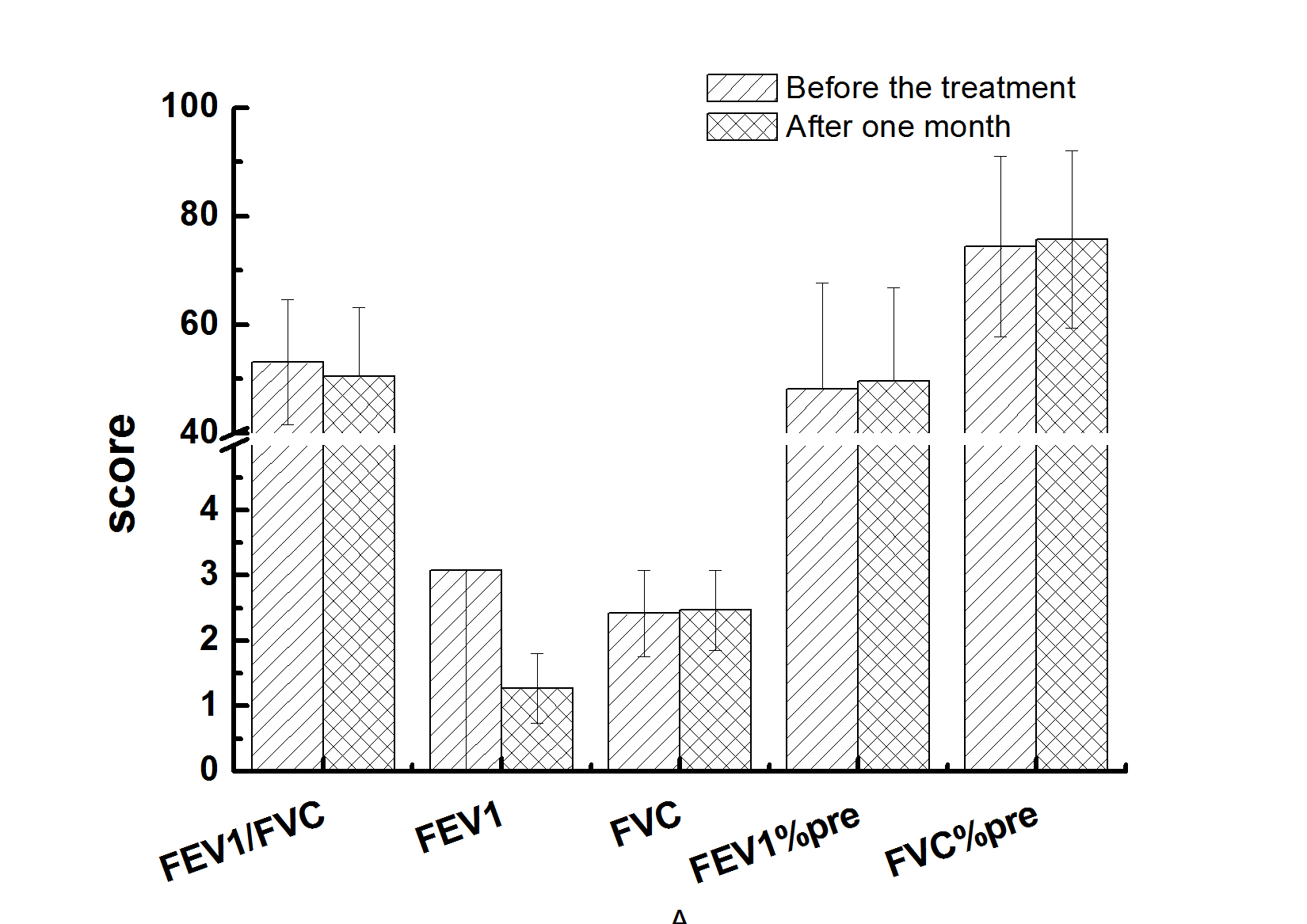

Supplement: Supplementary file 1 — Figure 1: Pulmonary functions in the two groups after one month of treatment. Figure 2: Pulmonary functions in the two groups after two months of treatment. Figure 3: Pulmonary functions in the two groups after three months of treatment. Figure 4: CAT, mMRC, and TCM syndrome scores in the two groups after one month. Figure 5: CAT, mMRC, and TCM syndrome scores in the two groups after two months of treatment. Figure 6: CAT, mMRC, and TCM syndrome scores in the two groups after three months of treatment. Figure 7: Pulmonary functions in the YQGB group after one month and before treatment. Figure 8: Pulmonary functions in the YQGB group after two months and before the treatment. Figure 9: Pulmonary functions in the YQGB group after three months and before the treatment. Figure 10: Pulmonary functions in the Pb group after one month and before the treatment. Figure 11: Pulmonary functions in the Pb group after two months and before the treatment. Figure 12: Pulmonary functions in the Pb group after three months and before the treatment. Figure 13: CAT and mMRC scores in the YQGB group after one month and before the treatment. Figure 14: CAT and mMRC in the Pb group after one month and before the treatment. Figure 15: CAT and mMRC in the YQGB group after two months and before the treatment. Figure 16: CAT and mMRC in the Pb group after two months and before the treatment. Figure 17: CAT and mMRC in the YQGB group after three months and before the treatment. Figure 18: CAT and mMRC in the Pb group after three months and before the treatment. [file 9130804.f1.zip › 图表数据/Figure7.jpg]

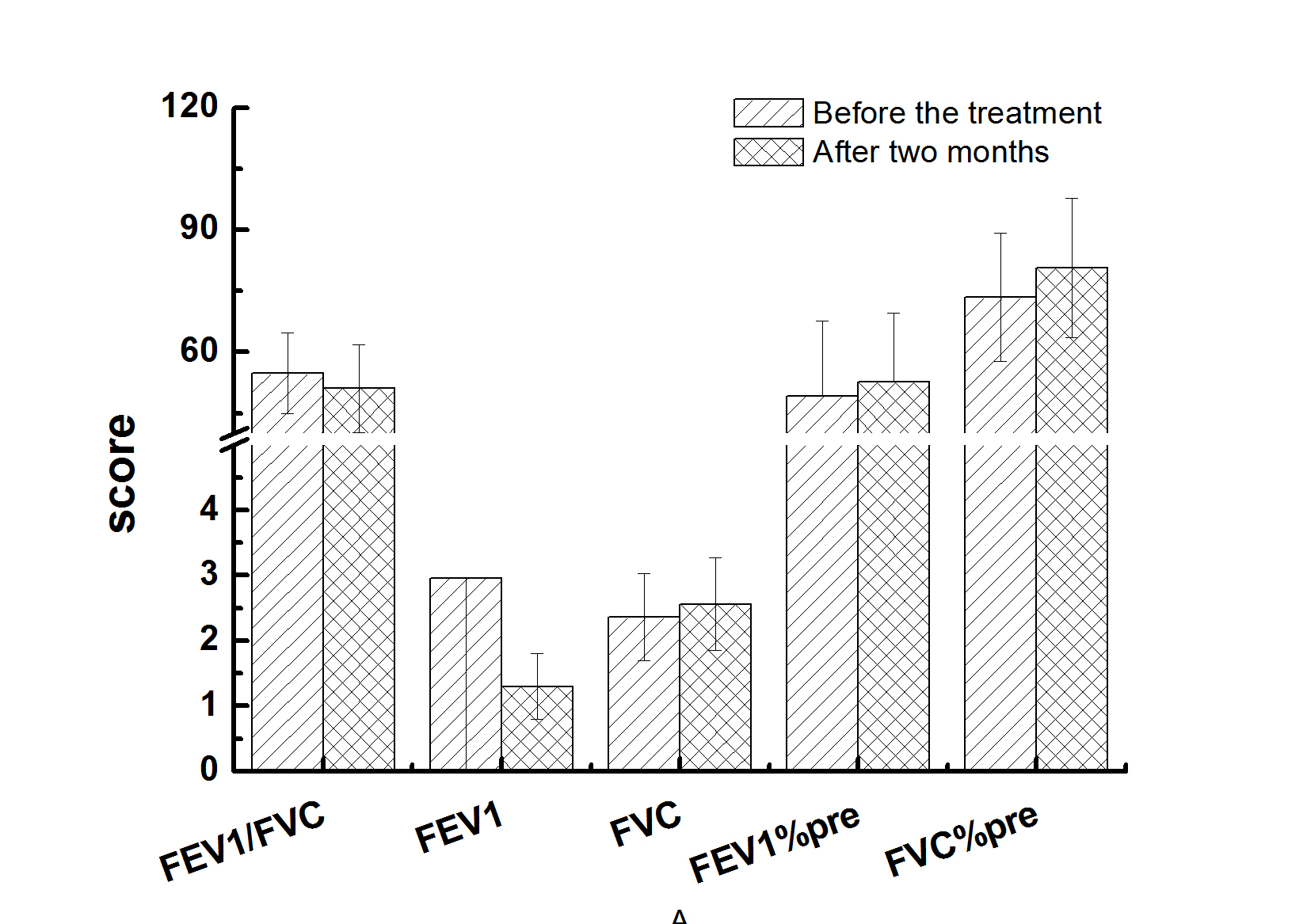

Supplement: Supplementary file 1 — Figure 1: Pulmonary functions in the two groups after one month of treatment. Figure 2: Pulmonary functions in the two groups after two months of treatment. Figure 3: Pulmonary functions in the two groups after three months of treatment. Figure 4: CAT, mMRC, and TCM syndrome scores in the two groups after one month. Figure 5: CAT, mMRC, and TCM syndrome scores in the two groups after two months of treatment. Figure 6: CAT, mMRC, and TCM syndrome scores in the two groups after three months of treatment. Figure 7: Pulmonary functions in the YQGB group after one month and before treatment. Figure 8: Pulmonary functions in the YQGB group after two months and before the treatment. Figure 9: Pulmonary functions in the YQGB group after three months and before the treatment. Figure 10: Pulmonary functions in the Pb group after one month and before the treatment. Figure 11: Pulmonary functions in the Pb group after two months and before the treatment. Figure 12: Pulmonary functions in the Pb group after three months and before the treatment. Figure 13: CAT and mMRC scores in the YQGB group after one month and before the treatment. Figure 14: CAT and mMRC in the Pb group after one month and before the treatment. Figure 15: CAT and mMRC in the YQGB group after two months and before the treatment. Figure 16: CAT and mMRC in the Pb group after two months and before the treatment. Figure 17: CAT and mMRC in the YQGB group after three months and before the treatment. Figure 18: CAT and mMRC in the Pb group after three months and before the treatment. [file 9130804.f1.zip › 图表数据/Figure8.jpg]

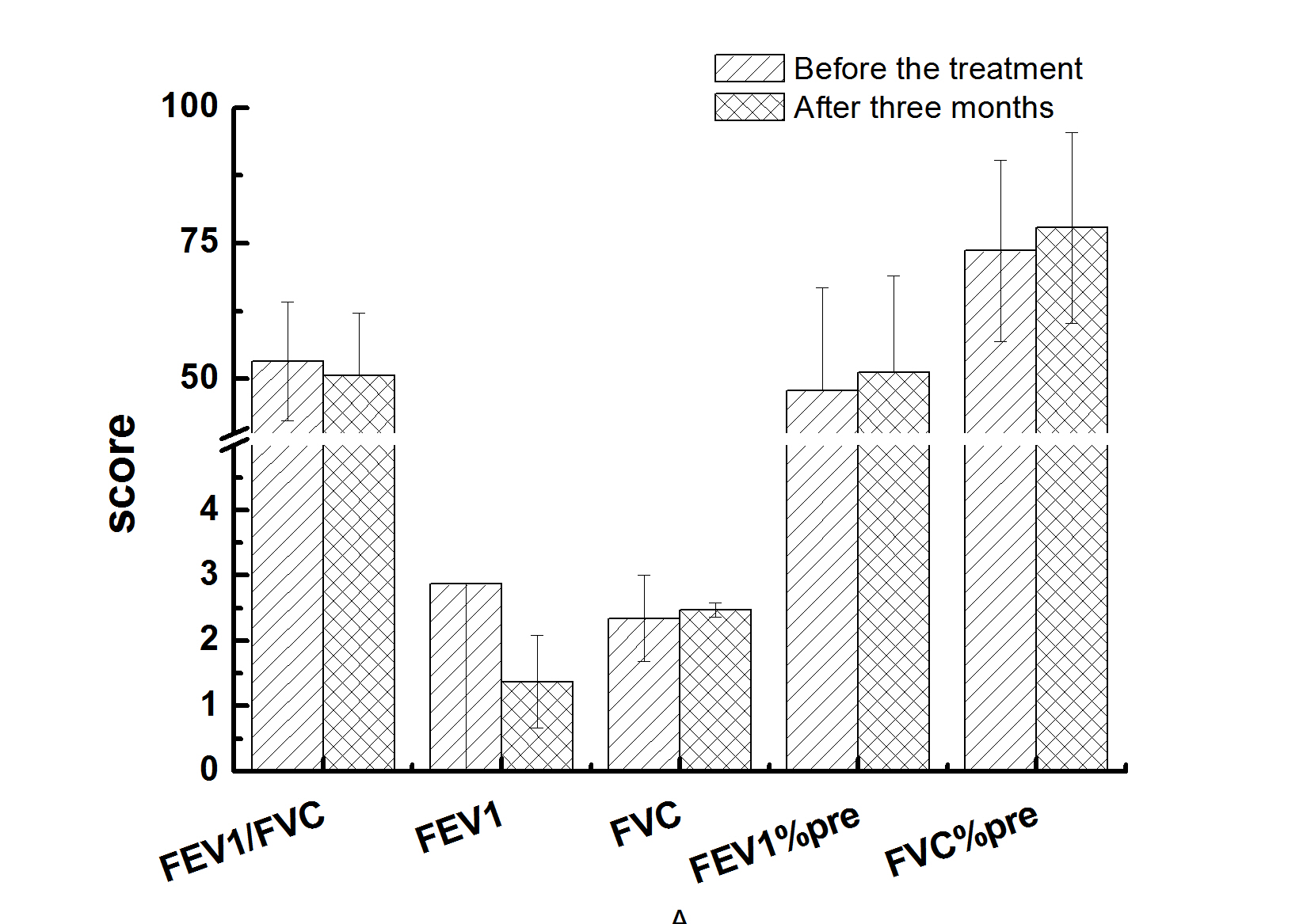

Supplement: Supplementary file 1 — Figure 1: Pulmonary functions in the two groups after one month of treatment. Figure 2: Pulmonary functions in the two groups after two months of treatment. Figure 3: Pulmonary functions in the two groups after three months of treatment. Figure 4: CAT, mMRC, and TCM syndrome scores in the two groups after one month. Figure 5: CAT, mMRC, and TCM syndrome scores in the two groups after two months of treatment. Figure 6: CAT, mMRC, and TCM syndrome scores in the two groups after three months of treatment. Figure 7: Pulmonary functions in the YQGB group after one month and before treatment. Figure 8: Pulmonary functions in the YQGB group after two months and before the treatment. Figure 9: Pulmonary functions in the YQGB group after three months and before the treatment. Figure 10: Pulmonary functions in the Pb group after one month and before the treatment. Figure 11: Pulmonary functions in the Pb group after two months and before the treatment. Figure 12: Pulmonary functions in the Pb group after three months and before the treatment. Figure 13: CAT and mMRC scores in the YQGB group after one month and before the treatment. Figure 14: CAT and mMRC in the Pb group after one month and before the treatment. Figure 15: CAT and mMRC in the YQGB group after two months and before the treatment. Figure 16: CAT and mMRC in the Pb group after two months and before the treatment. Figure 17: CAT and mMRC in the YQGB group after three months and before the treatment. Figure 18: CAT and mMRC in the Pb group after three months and before the treatment. [file 9130804.f1.zip › 图表数据/Figure9.jpg]
